# Analysis of the Antiproliferative Effect of Ankaferd Hemostat on Caco-2 Colon Cancer Cells via LC/MS Shotgun Proteomics Approach

**DOI:** 10.1155/2019/5268031

**Published:** 2019-05-21

**Authors:** Engin Koçak, Mustafa Çelebier, Ibrahim C. Haznedaroglu, Sacide Altınöz

**Affiliations:** ^1^Department of Analytical Chemistry, Faculty of Pharmacy, Hacettepe University, Ankara 06100, Turkey; ^2^Department of Hematology, Faculty of Medicine, Hacettepe University, Ankara 06100, Turkey

## Abstract

Ankaferd hemostat (ABS), a traditional herbal extract, is a hemostatic agent used for wound healing and bleeding treatment. A standardized form of plants contains many biomolecules. In recent years, previous studies have demonstrated the antineoplastic effect of ABS. In the present work, we focused on the mechanism of its antineoplastic effect over Caco-2 colon cancer cells. The LC/MS-based proteomics method was used to understand the effect of ABS at the protein level. The results were evaluated with gene ontology, protein interaction, and pathway analysis. As shown by our results, ABS altered glucose, fatty acids, and protein metabolism. Moreover, ABS affects the cell cycle machinery. Moreover, we found that ABS induced critical cancer target and suppressor proteins such as carboxyl-terminal hydrolase 1, 60S ribosomal protein L5, Tumor protein D52-like2, karyopherin alpha 2, and protein deglycase DJ-1. In conclusion, the proteomics results indicated that ABS affects various cancer targets and suppressor proteins. Moreover ABS has systematical effect on cell metabolism and cell cycle in Caco-2 cells, suggesting that it could be used as an antineoplastic agent.

## 1. Introduction

Ankaferd hemostat (Ankaferd blood stopper®, ABS) is a hemostatic agent that contains standardized plant extracts of* Thymus vulgaris*,* Glycyrrhiza glabra*,* Vitis vinifera*,* Alpinia officinarum*, and* Urtica dioica*. ABS is an endemic traditional formulation and has been widely used in Anatolia. By regulating protein network, ABS's main therapeutic effect is its hemostatic activity. ABS-stimulated modulation of erythroid proteins (ankyrin, spectrin, and actin) can lead to vital erythroid aggregation via acting on fibrinogen gamma. Randomized clinical studies have disclosed the efficiency and safety of ABS in clinical bleedings [1].

In recent years, ABS has drawn significant attention, with several studies conducted to observe its antimicrobial and antineoplastic effects [2–4]. As results demonstrated, ABS hemostat is extremely effective and has a high potential for treating cancer. ABS induces DNA damage by generating ROS production in cancer cells; and ABS's antiproliferative effects might depend on its prooxidant activity [5]. Dose-dependent studies showed that ABS inhibits the cell invasion of Saos-2 cancer cells. Moreover, the inhibition of cellular reproduction and decreased viability of human colon Caco-2 cells correlate with the applied ABS concentration* in vitro*. The cell invasion was also inhibited in a dose-dependent manner. It was observed that the Caco-2 cells which were exposed to ABS lost their adhesive characteristics in vitro and significant viability [3]. In another study, Gulec et al. indicated that ABS influences their mRNA expression of iron-regulated genes in Caco-2 and HepG-2 cells [6]. Likewise, Turhan et al. showed that topical ABS administration to the gastrointestinal neoplastic tissue resulted in the control of bleeding and decreased tumor vascularization for rectal and gastric cancers [7].

In fact, ABS's antineoplastic action was not an unexpected finding because ABS is a standardized form of four plants. Koluman et al. disclosed the antioxidant content of ABS with LC/MS [8]. ABS contains tocotrienols, vitamin E, tryptophan, estriol, galangin, apigenin, oenin, 3,4-divanillyltetrahydrofuran, TBHQ, thymol, BHA, BHT, lycopene, glycyrrhetinic acid, and tomatine. Most of these antioxidants have anticancer effects and, moreover, they could show synergic effects on neoplastic cells inside ABS. Moreover, bioactive compounds of ABS were investigated at protein level. Demiralp et al. showed that ABS contains important antineoplastic bioactive proteins (midkine, interleukin 4, p18, 18 kDa phosphoprotein, SUMO-protein ligase PIAS2, abhydrolase bundle transporter protein, the precursor of usher, tet oncogene family member 2 isoform b, twinfilin-1, SUMO-protein ligase PIAS2, prolactin secretor hormone receptor, protein phosphatase 1 regulatory subunit 12A, never in mitosis gene a (NIMA)-related kinase, mitochondrial protein, and mitochondrial actin binding protein 1) and they discussed these proteins were very important mode of action of ABS over tumors [9].

As a result, there have been expanding evidence regarding the antineoplastic effect of ABS; however, there is a lack of an in-depth study to elucidate its mechanism of action on cancer cells. As described previously, ABS contains many bioactive materials, and because of this pharmacobiological feature, it is expected that ABS could induce various cellular processes with different targets. Besides its antineoplastic effect, recent studies showed that ABS is a nontoxic plant mixture [10]. Its nontoxic feature allows it to be used on cancer patients safely as a food ingredient.

The LC/MS-based shotgun proteomics approach provides an opportunity to analyze hundreds of proteins, which are encoded in cells, to understand the cellular process at the protein level [11]. In this study, ABS's anticancer effect was evaluated by using the LC/MS-shotgun proteomics approach on Caco-2 cancer cells. Caco-2 cancer cells treated with ABS (named as group T) proteome level were compared with the one untreated with ABS (named as C group) in order to evaluate the therapeutic mechanism of ABS. The differentially expressed proteins were detected using the two-sample t-test. Gene ontology, protein interaction, and pathway analysis were used to evaluate altered proteins in cell biology.

A blood-stopper hemostatic agent, ABS, has been used for healing wounds. Likewise, as previous studies disclosed, ABS could be used for the controlling of bleeding within the gastrointestinal system [12–15]. This therapeutic intervention is especially important for the patients with colon and gastric cancer. During recent years, the anticancer effect of ABS has been well-studied and drawn attention for clinical use. Although numerous studies had been conducted about the anticancer effect of ABS, no detailed information regarding its therapeutic mechanism is currently available [7, 16, 17]. In this work, for the first time, the anticancer effect of ABS was assessed at the molecular level using LC/MS proteomics. The results showed that ABS induced cellular metabolisms such as glucose metabolism, cell cycle, and apoptosis processes. Moreover, in this study, we have dissected the resistant mechanism of Caco-2 cells against the pharmacobiological actions of ABS. This work will contribute to clarify ABS's anticancer effect on neoplastic cells.

## 2. Materials and Methods

### 2.1. Sample Preparation for Proteomic Analyzes

The cytosolic fractions of the ABS-treated and untreated Caco-2 cells were supplied from the TUBITAK project 114S500 (2014–2017). The Caco-2 cells were treated with ABS at a 10 *μ*L/mL (volume/volume) ratio for 48 hours or left untreated. ABS was supplied from commercial markets. The protein concentrations of cell lysates were determined by using the Bio-Rad DC (Bio-rad, USA) assay. The bovine serum albumin (BSA) was used as the standard. It was diluted with a homogenization buffer-water in a calibration range between 0.2 and 1.5 mg/ mL, while the collected samples were diluted with water (50:50 v/v). The absorbance of standards and samples was measured at 750 nm.

For the protein extraction from cell lysates, chloroform (sigma, USA)/methanol (sigma, USA)/water cosolvent system was used. A total of 400 uL methanol were added to 100 *μ*L cell lysates, followed by 100 *μ*L chloroform, and, finally, 300 *μ*L water was added to the mixtures. The precipitated proteins were suspended in a 100 mM Ammonium bicarbonate (sigma) solution, which contained 20% methanol (v/v). The proteins were reduced with 200 uM dithiothreitol (DTT) (sigma, USA) for one minute at 56°C and alkylated with 100 uM iodoacetamide (sigma, USA) for 30 minutes at room temperature. The proteins were digested by Trypsin (1:100 W/W) at 37°C for 16 hours incubation. The tryptic peptides were dissolved in acetonitrile that contained 0.1% formic acid (sigma).

### 2.2. LC-MS/MS Analysis

The peptide mixtures were separated in a C18 column (Zorbax C18 column 150 × 2.1 mm, 1.8 *μ*m, 300 Å (Agilent, USA)) by using Agilent HPLC-1290. The column temperature was maintained at 40°C, and the peptides were separated with a buffer system of 0.1% formic acid in water (A) and 0.1% formic acid in acetonitrile (B). The flow rate was adjusted to 0.200 *μ*L/min. The peptides were eluted with a gradient of 3%–50% mobile phase B over 130 minutes followed by 30%–90% mobile phase B over 10 minutes. An Agilent Q-TOF-MS 6530 system was used to analyze the peptides. Once separated, they passed through the electrospray ionization source (ESI) source and were ionized in the positive mode. The capillary voltage was adjusted at 4000 V with a drying temperature of 350°C. The auto MS-MS data were recorded between 300 and 1400 m/z above the 1500 count threshold. The MS-MS cycle time was 3.63s, and the maximum ion number was selected as three. The ion charge states were +2, +3, and +4, and the fragmentation energy was adjusted to 45 V. For each analysis, 80 *μ*g protein was loaded to the LC/MS system. Three technical replicates were analyzed for the treated and untreated groups.

### 2.3. Protein Identification and Quantification

For protein identification, the recorded MS-MS data were processed using the Maxquant. Homo sapiens database, which was downloaded from UniProtKB (https://www.uniprot.org/help/uniprotkb). The recorded MS-MS data were used to match with the in silico MS-MS data to identify peptides and proteins. In the matching process, 20 ppm of mass tolerance was used for the first and main search. Carbamidomethylation on cysteine, oxidation on methionine, and acetylation on -N units of proteins were chosen as fixed and variable modifications. Two missed cleavages were allowed. In the identification process, the false discovery rate (FDR) value was selected as 0.01 for reliable identification. The maxquant label free algorithm (MLFQ) was used for semiquantification between ABS-treated and untreated groups. In this algorithm, the protein intensities were calculated using peptide intensities.

For statistical data analysis, the R-based Perseus program, version 1.4.0.2, was used. The protein intensities were transformed to Log2 form, and a multiscatter plot analysis was used to observe the reproducibility of technical replicates with the Pearson correlation coefficient. The principal component analysis (PCA) was used to observe the differences between treated and untreated groups. Finally, to find significantly-expressed proteins, the two-sample t-test was performed.

### 2.4. Gene List, Protein-Protein Interaction, and Pathway Analyzes

The differently expressed proteins were evaluated in terms of their molecular biology. The PANTHER gene list analysis was used to evaluate the up- and downregulated proteins in terms of molecular functions and cellular components. For the gene list analysis, the Homo sapiens taxonomic unit was selected, and the official gene symbol was used. To analyze protein interactions, a STRING functional proteomic tool was used. The up- and downregulated proteins were analyzed separately to observe the relationship between them. The REACTOME pathway analysis was used to observe ABS-induced cellular pathways. 

## 3. Results

### 3.1. Protein Identification and Quantification

The tryptic peptides of the ABS-treated (Group T) and untreated groups (Group C) were analyzed using the LC/MS/MS system with three technical replicates. In the LC/MS/MS analyses, the average number of MS/MS was recorded as 5501 and 5499 for untreated and treated groups. The recorded MS/MS data were used in the matching process to identify peptide and protein. In the untreated group, the average number of identified peptides was 2085. This result showed that 37.8% of the MS/MS data matched the database. For the treated group, the average number of identified peptides was 2103, and its yield of matching was 38.2%. The median matching scores for the groups were 56 and 58.

**Figure 1:**
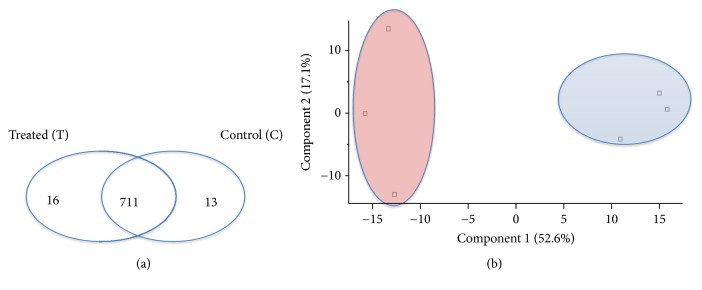
(a) Identified number of proteins in control and ABS treated groups. (b) PCA analysis of control and ABS groups. Blue zone shows technical replicates of control groups and red zone shows technical replicates of ABS treated group.

The recorded MS/MS data were processed with the Maxquant proteomics tool, and 727 and 724 proteins were identified by matching process for group T and C (Figure 1(a)). In each group, 711 proteins were identified (given at supplementary information (available here)). Identified proteins were also compared proteomics analysis of ABS in literature [9]. In present work, we did not find any plant-based ABS's protein.

The protein intensities were calculated using a label free quantification algorithm, and the results were processed using the Perseus statistic software (http://www.coxdocs.org/doku.php?id=perseus:start). The technical replicates of proteome measurements showed high quantification reproducibility for treated and untreated groups with Pearson correlation coefficients (multiscatter plot analysis) ranging from 0.987 to 0.994. The PCA was used to show the effects of Ankaferd on the overall proteome of Caco-2 cells and also similarity of technical replicates (Figure 1(b)). As the PCA analysis showed, the overall proteome structures of treated and untreated groups were different, and this difference was induced by ABS. Moreover, the analysis showed a high reproducibility for technical replicates in each group.

Finally, the two-sample t-test was used to find differentially expressed proteins. It was found that 163 proteins were differentially expressed between the groups. In supplementary information, all differentially expressed proteins were listed with fold changes.

### 3.2. Functional Classification Analysis

**Figure 2:**
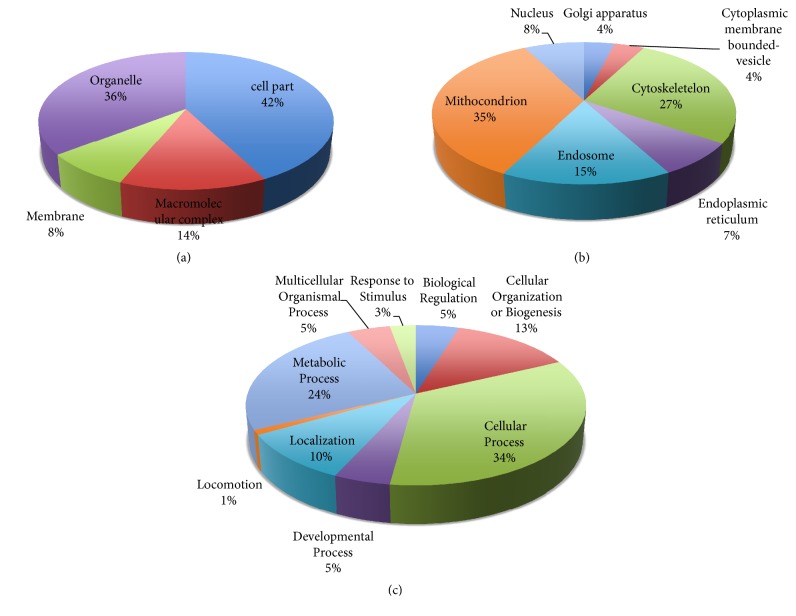
(a) Classification of downregulated proteins in cellular component. (b) Classification of downregulated proteins in organelles. (c) Classification of downregulated proteins in cellular processes.

Altered proteins were classified functionally by gene ontology analysis. We analyzed down- and upregulated proteins separately. Firstly we classified downregulated proteins according to cellular component and biological processes (Figure 2). We found that 42% of downregulated proteins are located in cellular part and 36% of proteins are in organelles (Figure 2(a)). We analyzed downregulated proteins in organelles and found most of them (35%) in mitochondrion (Figure 2(b)). In addition we classified proteins according to biological processes. We found that ABS affects cellular processes (34%) and metabolic processes (25%).

**Figure 3:**
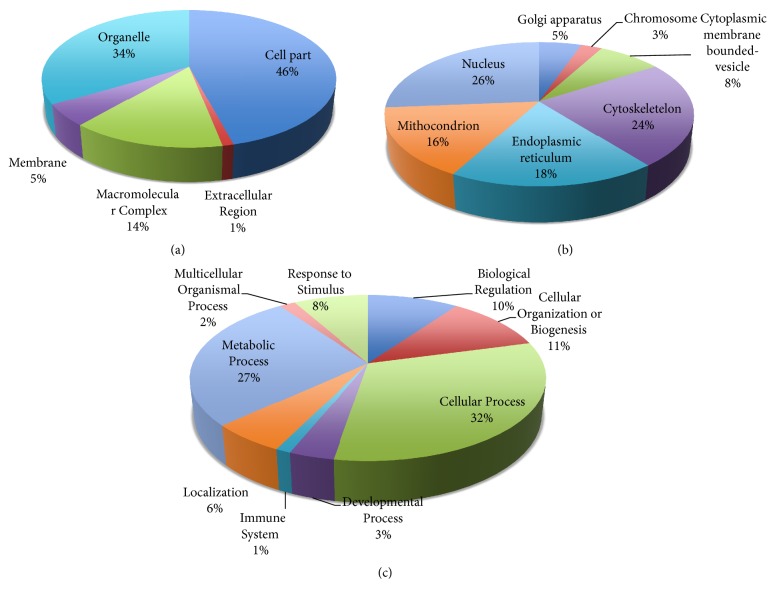
(a) Classification of upregulated proteins in cellular component. (b) Classification of upregulated proteins in organelles. (c) Classification of upregulated proteins in cellular processes.

Also upregulated proteins were classified with the same parameters (Figure 3). Most of the upregulated proteins are located in cell part (46%) and organelles (34%) as well as downregulated proteins (Figure 3(a)). We found that ABS affects many cellular component and biological processes (Figures 2(c) and 3(c)). As expected, profiles of down- and upregulated proteins in cell are different.

### 3.3. Protein Interaction Analysis

**Figure 4:**
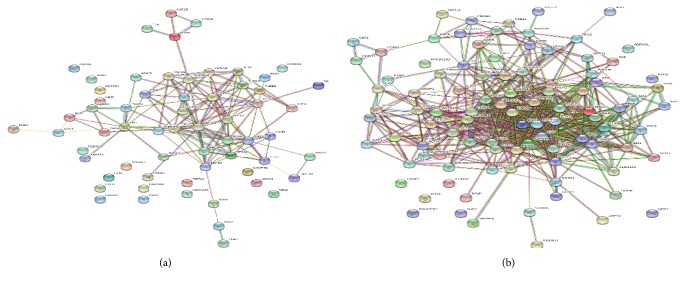
(a) Map of downregulated proteins. (b) Map of upregulated proteins.

In present work, we investigated relationship of differentially expressed proteins in string functional proteomics platform (Figure 4). We tried to observe systematic effect ABS over Caco-2 cells.

### 3.4. Protein Pathway Analysis

Altered proteins were investigated in pathway analysis. We found that ABS affects cellular metabolism and cell cycle machinery. ABS induces many important regulators in energy metabolism and fatty acid metabolism. Moreover, ABS affects cell cycle and apoptosis processes. In addition, effects of ABS on cancer targets and cancer suppressor proteins were analyzed.

## 4. Discussion

### 4.1. Protein Classification Analysis

The differently expressed proteins were evaluated using the PANTHER gene list analysis tool. First, the downregulated proteins were analyzed in the gene list. Many of them were found in the cell part and organelles in the cell (Figure 2(a)). When we analyzed the downregulated proteins more detailed in organelles, the mitochondrion proteins were observed to be induced by ABS. Since they are related to energy metabolism, it could be concluded that ABS affects the energy metabolism of the Caco-2 cell line. Several proteins were observed to be downregulated in the endoplasmic reticulum (ER) and Golgi apparatus (Figure 2(b)). As it is already known, the ER is the largest cell organelle and a major site of protein synthesis and transport, protein folding, lipid and steroid synthesis, carbohydrate metabolism, and calcium storage. The Golgi apparatus is responsible for transporting, modifying, and packaging proteins and lipids into vesicles for delivery to targeted destinations. It is located in the cytoplasm next to the ER and adjacent to the cell nucleus. As these results showed, ABS induced several pathways in macromolecule synthesis and transportation in cells.

Another point observed from the results was the downregulation of proteins, which are in vesicles bounded in the endosome and cytoplasmic membrane. Therefore, ABS was shown to be effective in cell trafficking and communication.

In Figure 2(c), the biological processes, in which downregulated proteins existed, are shown. Downregulated proteins by ABS, as results showed, were involved in cell cycle, cell communication, cellular component movement, and cytokinesis. Another important point in Figure 2(c) was the downregulation of proteins, which functions in metabolic processes. In addition, ABS reduced the expression level of proteins, which are involved in stimulus response.

The upregulated proteins were also evaluated in the gene list analysis. In Figure 3, upregulated proteins are shown in cellular component and biological process charts. In Figure 3(a), the cellular components, in which unregulated proteins were detected, are shown.  Figure 3(b) illustrates the upregulated proteins in organelles. The most interesting point was the overexpression of X-ray repair cross-complementing protein 5 in the chromosome. The XRCC5 heterodimer, which promotes cancer progression, binds the ends of broken DNA double strands to accomplish DNA nonhomologous end joining repair to maintain the stability of the whole genome and chromosomes. The overexpression of these proteins showed that ABS induced DNA damage and cancer cells tried to develop resistance against ABS's toxicity. In Figure 3(c), the cellular process, in which unregulated proteins are involved, is shown. As expected, we found two upregulated proteins TNF receptor-associated factor 3 (CAP1) and Heat shock 70 kDa protein 4 (HSPA4) related to the process of the immune system. As previous studies showed, these two proteins are crucial for cancer progression, stress response, and cell proliferation. Also, in Figure 3(c), we observed the overexpression of proteins, which is involved in drug-conditioned stimulus response. As the results showed, ABS has an antiproliferative effect on Caco-2 cells, and the cells tried to develop a protective response.

### 4.2. Protein-Protein Interaction

The protein-protein interaction networks are significant tools in understanding cellular processes such as metabolism, signal transduction, and drug resistance. In this work, the interactions of the identified proteins were mapped by searching the STRING database (https://string-db.org). In Figure 4(a), the downregulated proteins are shown in a protein map. As evident, many of the proteins, which were inhibited by ABS, are functionally linked: proteins were linked with several interactions, and the p value for protein-protein interaction enrichment was lower than 1e-16. This protein map and interactions showed that ABS affected not only individual targets but also the cellular process systematically.

Figure 4(b) depicts the interactions of upregulated proteins: upregulated proteins were linked to each other. The p value for protein-protein interaction enrichment was lower than 1e-16. The upregulated proteins were generally involved in cell defense and antioxidant proteins. These proteins were linked and worked together to protect the Caco-2 cells against the effect of ABS.

### 4.3. Pathway Analysis

To understand ABS's effect on cellular processes, a reactome pathway tool (https://reactome.org) was used to analyze the differently expressed proteins and find out the metabolic pathways affected by ABS.

### 4.4. Glucose Metabolism

The pathway analysis showed that ABS has a critical effect on glucose metabolism, which plays a key role in cancer progression. The clinical diagnosis of cancer using positron emission tomography relies on the accelerated rate of glucose metabolism, which is one of the metabolic signatures of cancer cells. In many studies, the Glycosis pathway was evaluated as one of the main targets for cancer treatment [18, 19]. As shown by the proteomics results, ABS reduced the expression level of ALDOA, PGAM1, and TPI1, which are involved in glycolysis pathway.

ALDOA is one of the key enzymes in glycolysis pathway; and, recently, ALDOA was discovered to be highly expressed in a variety of malignant cancers, including human lung cancer, osteosarcoma, colorectal cancer, oral squamous cell carcinomas, and hepatocellular carcinomas. It could serve as a diagnostic and prognostic biomarker [20]. Shurong et al. demonstrated that the silencing of the ALDOA expression inhibited the proliferation and invasion of PANC-1 cells. Kawai et al. showed that the ALDOA expression was negatively related to chemosensitivity and radiosensitivity and positively associated with proliferation [21].

The phosphoglycerate mutase 1 (PGAM1), which catalyzes the conversion of 3-phosphoglycerate into 2-phosphoglycerate, is another key enzyme in glycolysis [22]. In recent years, several studies have shown that PGAM1 levels are higher in several human cancers [23–25]. Evans et al. and Ren et al. have observed that PGAM1 plays an important role in tumor growth and the inhibition of PGAM1, which could cause the death of tumor cells [26, 27].

In the present study, it was observed that ABS forced the expression level of triosephosphate isomerase one protein (TPI1) in Caco-2 cells, which catalyzes the interconversion of dihydroxyacetone phosphate (DHAP) and d-glyceraldehyde-3-phosphate (G3P) during glycolysis and gluconeogenesis, and it is a crucial enzyme in carbohydrate metabolism. As several studies showed, TPI is highly expressed in tumors such as in esophageal cancer [28], colon cancer [29], and pancreatic cancer [30].

Hence, our results disclosed that ABS causes dysregulation in glucose metabolism by downregulation of ALDOA, PGAM1, and TPI1.

### 4.5. Pentose Phosphate Metabolism

A pentose-phosphate pathway is required for the synthesis of ribonucleotides, and it is a major source of NADPH. NADPH is essential for the scavenging of reactive oxygen species derived from oxidative phosphorylation, which is necessary for ATP generation. Thus, NADPH homeostasis is an important step in cancer progression. In recent years, the pentose-phosphate metabolism has emerged as a potential target for cancer therapy [31, 32]. The 6-phosphogluconate dehydrogenase (6PGD) is one of the key modulators in pentose-phosphate metabolism [33]. As studies showed, 6PGD is essential for cell proliferation and contributes to tumor growth [34]. Several studies have showed that the inhibition of 6PGD sensitizes cancer cells to oxidative stress induced by chemotherapeutic agents [35]. In the present work, we observed that ABS downregulated 6PGD and inhibited the pentose-phosphate metabolism. ABS probably sensitized the Caco-2 cells to oxidative stress.

### 4.6. Fatty Acid Metabolism

In the present work, it was observed that ABS affected the fatty acid metabolism in Caco-2 cells. Fatty Acid Synthase (FASN) and Acetyl-CoA Acetyltransferase 2 (ACAT2), the key proteins in fatty acid metabolism, were found to be downregulated in the group treated with ABS. Fatty acid metabolism plays an important role in providing energy, macromolecules for membrane synthesis, and lipid signals during cancer development [36]. In recent years, fatty acid metabolism has been evaluated as a potential cancer target for treatment, and, according to the results, the inhibition of FASN and ACAT2 leads cancer cells to apoptosis [37, 38].

### 4.7. Protein Metabolism

Cancer cells are inherently capable of reprograming the cellular proteome to adapt to a stressful tumor microenvironment. Protein translation is one of the key steps for the reprograming of proteome and contributes to the progress and development of cancer [39]. The overexpression of eukaryotic translation elongation factors, which are the main regulators in protein metabolism and translation, promotes cancer development [40, 41]. In recent years, the protein translation process has emerged as a potential new target and a key process to develop new innovative treatments for cancer [42, 43]. As the proteomics results showed, ABS has a systematic effect on protein translation. It especially affected and decreased the expression level of elongation factor proteins (EEF1D, EEF1A1, EEF1B2, EEF1G, EIF3M, and EEF2), which are the main regulators in protein translation. ABS, therefore, has a significant impact on protein metabolism and synthesis.

### 4.8. Impact of ABS on Cell Cycle and Apoptosis

Cancer is characterized by the proliferation of uncontrolled tumor cell resulting from the aberrant activity of various cell cycle proteins. The origin of cancer depends on the uncontrolled cell cycle process and is sensitive to their inhibition. In recent years, cell cycle regulators have emerged as an attractive and potential target for cancer treatment.

In our experiments, we observed that 26S proteasome non-ATPase regulatory subunit 1 (PMSD1), 14-3-3-protein zeta/delta (YWHAZ), and14-3-3 gamma (YWHAG), which were involved in cell cycle, were downregulated by inducing ABS.

In the literature, several previous studies suggested that YWHAZ is one of the cell cycle checkpoints and has an important role in various types of cancer. Nishumora et al. showed that YWHAZ has a pivotal role in tumor cell proliferation and its overexpression could be used as a prognostic biomarker. They observed that the knockdown of YWHAZ inhibits cell proliferation, migration, and invasion of gastric cancer cells [44] As Hong et al. observed, the YWHAZ downregulation of YWHAZ inhibited cell cycle progression, migration, and the expression of stem cell markers. Moreover, tumorigenicity was suppressed in tumor-bearing BALB/c nude mice following the YWHAZ knockdown [45].

YWHAG is a multifunctional protein involved in cell cycle regulation as a checkpoint, protein trafficking, and apoptosis. The overexpression of YWHAG has been observed in various cancer types [46–48]. Kim et al. showed that YWHAG inhibits apoptotic cell death and promotes cell migration in breast cancer [49]. In the present study, the results suggested that ABS significantly inhibited the YWHAG expression.

PMSD1 is another checkpoint regulator protein induced by ABS. In 2018, it was found that PMSD1 regulates cell growth, and the knockdown of PSMD1 exhibited cell cycle arrest and the accumulation of p53 protein through inhibiting p53 protein degradation in breast cancer [50]. All of these results showed that ABS affects the critical cell cycle points and leads to cell death.

### 4.9. Impact of ABS on Various Cancer Targets

In our present results, we observed that ABS is extremely effective on cell metabolism and cell cycle machinery. In addition, we evaluated potential cancer targets, which were induced by ABS.

In our present work, ABS was observed to decrease tumor protein D52-like 2 (TPD52L2), a protein which was assessed in several previous studies. It has been observed that TPD52L2 was involved in cell proliferation and was used as a prognostic biomarker [50, 51]. As the investigations indicated, TPD52L2 is a regulator in cell cycle and played an essential role in the cell growth for many cancer types. Wang et al. showed that the knockdown of TPD52L2 in ZR-75-30 cells increased the cell percentage of the G0/G1 phase and decreased the cell populations in the S-phase and G2/M phase, which could contribute to the inhibition of cell proliferation [52]. Likewise, Chi et al. showed that the inhibition of TPD52L2 suppressed cell proliferation and colony formation of prostate cancer cells [53]. Decreasing of TPD52L2 by ABS was a sign of ABS's effect on cell cycle and cell proliferation.

Another protein, karyopherin alpha 2 (KPNA2), which involves the nucleocytoplasmic transport process, was induced by ABS. In recent years, KPNA2 has emerged as a biomarker and a possible target for the treatment of various types of cancer [54, 55]. Huang et al. showed that KPNA2 promotes the migration and invasion of cancer cells, demonstrating that the silencing of KPNA2 could inhibit cell growth and survival [55]. Previous studies also dissected the molecular mechanisms of the effect of KPNA2-induced promotion of G1/S cell cycle transition. ABS inhibited KPNA2 expression and probably affected the G1/S cell cycle transition.

The staphylococcal nuclease and tudor domain containing 1 (SND1) is a multifunctional protein. SND1 is involved in the regulation of gene expression processes such as transcriptional activation, RNA splicing, editing, and stability and is vital for cell viability [56]. In recent years, SND1 has emerged as one of the most important modulators of the molecular network in cancer cells and a molecular target for cancer treatment. Moreover, SND1 was found to play important roles in stress conditions [57–59]. Jarilawa et al. showed that SND1 was a potential target for cancer treatment. In our present results, ABS was observed to decrease the expression level of SND1, and this result was critical for the elucidation of the anticancer effect of ABS. SND1 is an important cell viability regulator, and the inhibition of this target could affect various pathways in cellular processes. Further studies will shed light on SND1.

The expression of protein deglycase DJ-1 (PARK7), which was induced by ABS, was observed to be decreased. PARK7, originally identified as a novel oncogene [60], protects cancer cells against oxidative stress, mediating cell survival and proliferation by activating the extracellular signal-regulated kinase (ERK1/2) pathway, and attenuating cell death signaling by inhibiting apoptosis signal-regulating kinase 1 (ASK1) activation [61]. The PARK7 expression in colorectal cancer tissues was higher than that in normal colon tissues and PARK7 downregulated the tumor suppressor PTEN [62].

### 4.10. Impact of ABS on Cancer Suppressor Proteins

In our present work, the proteomics results showed that ABS induces the expression level of several cancer suppressor proteins. We evaluated those proteins in terms of cancer progression.

We observed that the expression level of ubiquitin carboxyl-terminal hydrolase 1 (UCHL1) is higher in ABS-treated groups. Several previous studies showed that UCHL1 is a tumor suppressor in several cancer cells. Jin et al. disclosed that the inhibition of UCHL1 promoted cell proliferation by increasing cells in S-phase and that the knockdown of UCHL1 reduced cell apoptosis and contributed to cisplatin resistance [63]. Moreover, Li et al. showed that UCHL1 promotes tumor suppressor p53 signaling and is silenced due to its promotion of methylation in nasopharyngeal carcinoma [64]. The upregulation of UCHL1 induced by ABS showed another effect of ABS on cell cycle machinery with different targets and mechanism.

Moreover, our proteomics results showed that 60S ribosomal protein L5 (RPL5) was upregulated in the ABS-treated group. RPL5 is one of the activators of p53 following ribosome biogenesis stress. Many cell cycle regulators regulate ribosome biogenesis, a key element in cell growth, and it is an important cancer target since the impairment of ribosome biogenesis, which is called ribosome biogenesis stress and leads to the activation of p53 and apoptosis. As studies revealed, several ribosomal proteins (RPL5 and RPL10) bind directly to MDM2 and activate the p53 apoptotic pathway upon ribosomal biogenesis stress [65, 66]. This result suggests that ABS affects the ribosome biogenesis and the activation of the p53 apoptotic pathway. Our present findings that elucidate the interactions between ABS and p53 are compatible with the previous* in vitro* transcriptomics and* in vivo* data that showed that ABS could activate the p53 pathway [67–69]. In further studies we will investigate the ABS-p53 pathway relationships with targeted proteomics and genomics experiments.

### 4.11. Caco-2 Resistance Mechanism to ABS

In our present experiments, we observed that ABS induced several critical pathways and proteins, and it is implicated as a significant anticancer activity. Moreover, we observed that the Caco-2 cells tried to gain resistance against ABS-induced cytotoxicity.

As our results showed, several heat shock proteins (HSPs) were altered in the ABS-treated groups. HSPs are stress proteins, of which synthesis is triggered by proteotoxic stresses. Those proteins are molecular chaperones and involved in the various biological processes such as synthesis and folding of proteins, secretion, trafficking, protein degradation, and regulation of transcription factors [70]. HSPs are often highly expressed in cancer cells [71]. They are very important in responding to stress conditions and promote cell survival in various conditions [72]. In our present experiments, we observed that HSPA9, HSPD1, HSPE1, HSP90B1, HSP70 (HSPA1B), HSPA5, and HSP47 (SERPINH1) were expressed highly in the ABS-treated groups. The HSP70 and HSP 90 expressions especially correlate with increased cell proliferation and resistance to chemotherapeutics.

The peroxiredoxin (PRDX) family is one of the most important antioxidant protein classes in the defense against oxidative stress [73]. In recent years, a large body of evidence has suggested their involvement in carcinogenesis and the development of drug resistance. Increasing the expression levels of PRDX family proteins, as studies showed, contribute to chemotherapeutic resistance. In the present study, it was observed that PRDX1, PRDX2, PRDX5, and PRDX6 proteins were upregulated in ABS-treated groups.

Glutathione S-transferases (GSTs) are a class of detoxification enzymes that catalyze the conjugation of potentially damaging chemical mutagens to glutathione and protect against the products of oxidative stress. Therefore, they are considered the most important phase II metabolizing enzyme. In our present experiments, we observed that the GSTP1 and GSTO1 proteins were upregulated in the ABS-treated group. Previous studies showed that the expression level of GSTP1 and GSTO correlates with oxidative stress. They also demonstrated that GSTO1 and GSTP1 protect cancer cells against oxidative stress.

In further experiments we will focus on the antineoplastic effect of ABS over other colon cancer cell lines like HT29 to compare our present findings, since sometimes fixing issues in cell culture experiments could cast some doubts to assess drug-cell interactions in traditional cancer research [74] and also any immortalized cell line and, in particular, colon cancer cell lines are artificial to some extent. To overcome any fixing problem in cell culture and improve our understanding of antineoplastic effect of ABS our research team will continue our experiments to further evaluate ABS with other cell lines.

## 5. Conclusion

The anticancer effects of ABS on Caco-2 cells have been known for a long time, but its therapeutic mechanism has not been clarified yet. In this work, we use LC/MS shotgun proteomics to understand the impact of ABS on Caco-2 cells. The results were processed with gene list analysis, protein-protein interaction, and reactome pathway analysis.

As our results showed, ABS significantly affects cell metabolism and cell cycle machinery. These results could be named as the main antiproliferative effects of ABS on cancer cells. Moreover, ABS affects the potential targets on cancer therapy such as SND1, KPNA2, and PARK7. Another critical finding was the upregulation of tumor suppressor proteins UCHL1 and RPL5. RPL5 especially, which is an activator of ribosome biogenesis stress, directly activates the p53 apoptotic pathway and causes apoptosis.

Moreover, we observed that heat shock, peroxidoxins, and GSTs family proteins were unregulated to protect against ABS's toxicity and develop resistance.

In conclusion, ABS, which contains various plant extracts, affects critical cellular processes. Further studies, metabolomics, genomics, and targeted proteomics will expand our work for an even better understanding of ABS-induced antineoplastic processes in a wide variety of cancers.

## Data Availability

The data used to support the findings of this study are available from the corresponding author upon request.

## Supplementary Materials

Supplementary Materials Table S1. In proteomics analysis of Caco-2 cells, 711 proteins were identified in control and Ankaferd (ABS) treated groups. The identified proteins with their names and gene symbols are presented in Table 1. Table S2. The list of down- and upregulated proteins under ABS stress with their gene symbols and fold changes (p<0.05)
