# Supplementary material for: Analysis of the Antiproliferative Effect of Ankaferd Hemostat on Caco-2 Colon Cancer Cells via LC/MS Shotgun Proteomics Approach
Source: Biomed Res Int. 2019 May 21;2019:5268031. doi: 10.1155/2019/5268031 (PMC6556321; doi:10.1155/2019/5268031)
Supplement: Supplementary Materials — Table S1. In proteomics analysis of Caco-2 cells, 711 proteins were identified in control and Ankaferd (ABS) treated groups. The identified proteins with their names and gene symbols are presented in Table 1. Table S2. The list of down- and upregulated proteins under ABS stress with their gene symbols and fold changes (p<0.05) [file 5268031.f1.zip › 5268031.f1/Table S1. Supplementary Material Description_BMRI_2767460.docx]

**Table S1. Supplementary Material Description**

In proteomics analysis of Caco-2 cells, 711 proteins were identified in control and Ankaferd (ABS) treated groups. The identified proteins with their names and gene symbols are presented in Table 2.

**Table S1.** Identified 711 proteins with their names and gene symbols.

| **No** | **Protein names** | **Gene symbols** |
| --- | --- | --- |
| 1 | 1-phosphatidylinositol 3-phosphate 5-kinase | PIKFYVE |
| 2 | 1-phosphatidylinositol 4,5-bisphosphate phosphodiesterase beta-2 | PLCB2 |
| 3 | 1-phosphatidylinositol 4,5-bisphosphate phosphodiesterase eta-1 | PLCH1 |
| 4 | 10 kDa heat shock protein, mitochondrial | HSPE1 |
| 5 | 14-3-3 protein beta/alpha;14-3-3 protein beta/alpha, N-terminally processed | YWHAB |
| 6 | 14-3-3 protein epsilon | YWHAE |
| 7 | 14-3-3 protein gamma;14-3-3 protein gamma, N-terminally processed | YWHAG |
| 8 | 14-3-3 protein theta | YWHAQ |
| 9 | 14-3-3 protein zeta/delta | YWHAZ |
| 10 | 26S proteasome non-ATPase regulatory subunit 1 | PSMD1 |
| 11 | 26S proteasome non-ATPase regulatory subunit 13 | PSMD13 |
| 12 | 26S proteasome non-ATPase regulatory subunit 2 | PSMD2 |
| 13 | 26S proteasome non-ATPase regulatory subunit 3 | PSMD3 |
| 14 | 40S ribosomal protein S12 | RPS12 |
| 15 | 40S ribosomal protein S9 | RPS9 |
| 16 | 40S ribosomal protein SA | RPSA |
| 17 | 6-phosphogluconate dehydrogenase, decarboxylating | PGD |
| 18 | 60 kDa heat shock protein, mitochondrial | HSPD1 |
| 19 | 60S acidic ribosomal protein P2 | RPLP2 |
| 20 | 60S ribosomal protein L5 | RPL5 |
| 21 | 78 kDa glucose-regulated protein | HSPA5 |
| 22 | A disintegrin and metalloproteinase with thrombospondin motifs 12 | ADAMTS12 |
| 23 | A disintegrin and metalloproteinase with thrombospondin motifs 2 | ADAMTS2 |
| 24 | A-kinase anchor protein 9 | AKAP9 |
| 25 | Abnormal spindle-like microcephaly-associated protein | ASPM |
| 26 | Acetyl-CoA acetyltransferase, cytosolic | ACAT2 |
| 27 | Acidic leucine-rich nuclear phosphoprotein 32 family member A | ANP32A |
| 28 | Acidic leucine-rich nuclear phosphoprotein 32 family member B | ANP32B |
| 29 | Actin filament-associated protein 1 | AFAP1 |
| 30 | Actin-related protein 2 | ACTR2 |
| 31 | Actin-related protein 3 | ACTR3 |
| 32 | Actin, alpha cardiac muscle 1;Actin, alpha skeletal muscle | ACTC1;ACTA1 |
| 33 | Actin, cytoplasmic 1;Actin, cytoplasmic 1, N-terminally processed | ACTB |
| 34 | Actin, cytoplasmic 2;Actin, cytoplasmic 2, N-terminally processed | ACTG1 |
| 35 | Activating signal cointegrator 1 complex subunit 3 | ASCC3 |
| 36 | Acylamino-acid-releasing enzyme | APEH |
| 37 | Adapter protein CIKS | TRAF3IP2 |
| 38 | Adenine phosphoribosyltransferase | APRT |
| 39 | Adenomatous polyposis coli protein | APC |
| 40 | Adenomatous polyposis coli protein 2 | APC2 |
| 41 | Adenylate cyclase type 10 | ADCY10 |
| 42 | Adenylosuccinate lyase | ADSL |
| 43 | Adenylyl cyclase-associated protein 1 | CAP1 |
| 44 | ADP-ribosylation factor 1;ADP-ribosylation factor 3 | ARF1;ARF3 |
| 45 | ADP-ribosylation factor 4 | ARF4 |
| 46 | Alanine--tRNA ligase, cytoplasmic | AARS |
| 47 | Aldo-keto reductase family 1 member C3 | AKR1C3 |
| 48 | Alpha-actinin-1 | ACTN1 |
| 49 | Alpha-actinin-3 | ACTN3 |
| 50 | Alpha-actinin-4 | ACTN4 |
| 51 | Alpha-enolase | ENO1 |
| 52 | Alpha-mannosidase 2 | MAN2A1 |
| 53 | Ankyrin repeat domain-containing protein 26 | ANKRD26 |
| 54 | Ankyrin repeat domain-containing protein 30B | ANKRD30B |
| 55 | Annexin A2 | ANXA2 |
| 56 | Annexin A3 | ANXA3 |
| 57 | Annexin A4 | ANXA4 |
| 58 | Annexin A5 | ANXA5 |
| 59 | Anterior gradient protein 2 homolog | AGR2 |
| 60 | Apolipoprotein B-100;Apolipoprotein B-48 | APOB |
| 61 | Apoptotic chromatin condensation inducer in the nucleus | ACIN1 |
| 62 | Arf-GAP with Rho-GAP domain, ANK repeat and PH domain-containing protein 2 | ARAP2 |
| 63 | Armadillo repeat-containing protein 2 | ARMC2 |
| 64 | Armadillo repeat-containing protein 4 | ARMC4 |
| 65 | Aromatase | CYP19A1 |
| 66 | Asparagine synthetase [glutamine-hydrolyzing] | ASNS |
| 67 | Aspartate aminotransferase, cytoplasmic | GOT1 |
| 68 | AT-rich interactive domain-containing protein 1A | ARID1A |
| 69 | Ataxin-7 | ATXN7 |
| 70 | ATP-binding cassette sub-family A member 12 | ABCA12 |
| 71 | ATP-citrate synthase | ACLY |
| 72 | ATP-dependent 6-phosphofructokinase, platelet type | PFKP |
| 73 | ATP-dependent RNA helicase DDX39A;Spliceosome RNA helicase DDX39B | DDX39A;DDX39B |
| 74 | Autophagy-related protein 2 homolog B | ATG2B |
| 75 | Band 4.1-like protein 4A | EPB41L4A |
| 76 | Beta-actin-like protein 2 | ACTBL2 |
| 77 | Bifunctional purine biosynthesis protein PURH;Phosphoribosylaminoimidazolecarboxamide formyltransferase;IMP cyclohydrolase | ATIC |
| 78 | Biorientation of chromosomes in cell division protein 1-like 1 | BOD1L1 |
| 79 | Bone morphogenetic protein 2 | BMP2 |
| 80 | Breast cancer type 2 susceptibility protein | BRCA2 |
| 81 | Brefeldin A-inhibited guanine nucleotide-exchange protein 2 | ARFGEF2 |
| 82 | Brefeldin A-inhibited guanine nucleotide-exchange protein 3 | ARFGEF3 |
| 83 | C-Jun-amino-terminal kinase-interacting protein 4 | SPAG9 |
| 84 | C-myc promoter-binding protein | DENND4A |
| 85 | CAD protein;Glutamine-dependent carbamoyl-phosphate synthase;Aspartate carbamoyltransferase;Dihydroorotase | CAD |
| 86 | Cadherin EGF LAG seven-pass G-type receptor 2 | CELSR2 |
| 87 | Cadherin EGF LAG seven-pass G-type receptor 3 | CELSR3 |
| 88 | Cadherin-like and PC-esterase domain-containing protein 1 | CPED1 |
| 89 | Calcium/calmodulin-dependent protein kinase type II subunit gamma | CAMK2G |
| 90 | Caldesmon | CALD1 |
| 91 | Calmodulin | CALM1 |
| 92 | Calreticulin | CALR |
| 93 | CAP-Gly domain-containing linker protein 1 | CLIP1 |
| 94 | Cardiomyopathy-associated protein 5 | CMYA5 |
| 95 | Cartilage intermediate layer protein 1;Cartilage intermediate layer protein 1 C1;Cartilage intermediate layer protein 1 C2 | CILP |
| 96 | CASP8-associated protein 2 | CASP8AP2 |
| 97 | CBP80/20-dependent translation initiation factor | CTIF |
| 98 | CCR4-NOT transcription complex subunit 1 | CNOT1 |
| 99 | Cell migration-inducing and hyaluronan-binding protein | CEMIP |
| 100 | Centlein | CNTLN |
| 101 | Centromere protein F | CENPF |
| 102 | Centrosomal protein of 120 kDa | CEP120 |
| 103 | Centrosomal protein of 152 kDa | CEP152 |
| 104 | Centrosomal protein of 170 kDa | CEP170 |
| 105 | Centrosomal protein of 290 kDa | CEP290 |
| 106 | Centrosomal protein of 295 kDa | CEP295 |
| 107 | Charged multivesicular body protein 4b | CHMP4B |
| 108 | Chloride intracellular channel protein 1 | CLIC1 |
| 109 | Chromatin assembly factor 1 subunit A | CHAF1A |
| 110 | Chromobox protein homolog 3 | CBX3 |
| 111 | Chromodomain-helicase-DNA-binding protein 1-like | CHD1L |
| 112 | Chromodomain-helicase-DNA-binding protein 7 | CHD7 |
| 113 | Chromodomain-helicase-DNA-binding protein 9 | CHD9 |
| 114 | Cilia- and flagella-associated protein 54 | CFAP54 |
| 115 | Cilia- and flagella-associated protein 69 | CFAP69 |
| 116 | Cilia- and flagella-associated protein 74 | CFAP74 |
| 117 | Citron Rho-interacting kinase | CIT |
| 118 | Clathrin heavy chain 1 | CLTC |
| 119 | Coagulation factor VIII;Factor VIIIa heavy chain, 200 kDa isoform;Factor VIIIa heavy chain, 92 kDa isoform;Factor VIII B chain;Factor VIIIa light chain | F8 |
| 120 | Coatomer subunit beta | COPB1 |
| 121 | Coatomer subunit gamma-1 | COPG1 |
| 122 | Cofilin-1 | CFL1 |
| 123 | Coiled-coil domain-containing protein 144A | CCDC144A |
| 124 | Coiled-coil domain-containing protein 151 | CCDC151 |
| 125 | Coiled-coil domain-containing protein 162 | CCDC162P |
| 126 | Coiled-coil domain-containing protein 177 | CCDC177 |
| 127 | Coiled-coil domain-containing protein 74A | CCDC74A |
| 128 | Coiled-coil domain-containing protein 93 | CCDC93 |
| 129 | Collagen alpha-1(XI) chain | COL11A1 |
| 130 | Collagen alpha-1(XII) chain | COL12A1 |
| 131 | Collagen alpha-3(VI) chain | COL6A3 |
| 132 | Collagen and calcium-binding EGF domain-containing protein 1 | CCBE1 |
| 133 | Complement C4-B;Complement C4 beta chain;Complement C4-B alpha chain;C4a anaphylatoxin;C4b-B;C4d-B;Complement C4 gamma chain;Complement C4-A;Complement C4 beta chain;Complement C4-A alpha chain;C4a anaphylatoxin;C4b-A;C4d-A;Complement C4 gamma chain | C4B;C4A |
| 134 | Complement receptor type 1 | CR1 |
| 135 | Condensin-2 complex subunit G2 | NCAPG2 |
| 136 | Cone cGMP-specific 3,5-cyclic phosphodiesterase subunit alpha | PDE6C |
| 137 | Copine-1 | CPNE1 |
| 138 | Costars family protein ABRACL | ABRACL |
| 139 | Creatine kinase B-type | CKB |
| 140 | Cullin-4A | CUL4A |
| 141 | Cullin-9 | CUL9 |
| 142 | Cyclin-dependent kinase 1 | CDK1 |
| 143 | Cyclin-dependent kinase-like 5 | CDKL5 |
| 144 | Cytoplasmic dynein 1 heavy chain 1 | DYNC1H1 |
| 145 | Cytoplasmic dynein 2 heavy chain 1 | DYNC2H1 |
| 146 | Cytoplasmic FMR1-interacting protein 2 | CYFIP2 |
| 147 | D-3-phosphoglycerate dehydrogenase | PHGDH |
| 148 | dCTP pyrophosphatase 1 | DCTPP1 |
| 149 | Dedicator of cytokinesis protein 4 | DOCK4 |
| 150 | DENN domain-containing protein 4B | DENND4B |
| 151 | Destrin | DSTN |
| 152 | Deubiquitinating protein VCIP135 | VCPIP1 |
| 153 | Dihydrolipoyllysine-residue acetyltransferase component of pyruvate dehydrogenase complex, mitochondrial | DLAT |
| 154 | Dihydropyrimidinase-related protein 2 | DPYSL2 |
| 155 | DmX-like protein 1 | DMXL1 |
| 156 | DNA (cytosine-5)-methyltransferase 3A | DNMT3A |
| 157 | DNA annealing helicase and endonuclease ZRANB3;DNA annealing helicase ZRANB3;Endonuclease ZRANB3 | ZRANB3 |
| 158 | DNA fragmentation factor subunit alpha | DFFA |
| 159 | DNA ligase 1 | LIG1 |
| 160 | DNA ligase 4 | LIG4 |
| 161 | DNA polymerase delta catalytic subunit | POLD1 |
| 162 | DNA replication licensing factor MCM7 | MCM7 |
| 163 | DNA-binding protein SATB2 | SATB2 |
| 164 | DNA-dependent protein kinase catalytic subunit | PRKDC |
| 165 | DnaJ homolog subfamily C member 13 | DNAJC13 |
| 166 | Down syndrome cell adhesion molecule-like protein 1 | DSCAML1 |
| 167 | Dynein heavy chain 1, axonemal | DNAH1 |
| 168 | Dynein heavy chain 10, axonemal | DNAH10 |
| 169 | Dynein heavy chain 14, axonemal | DNAH14 |
| 170 | Dynein heavy chain 2, axonemal | DNAH2 |
| 171 | Dynein heavy chain 5, axonemal | DNAH5 |
| 172 | Dynein heavy chain 6, axonemal | DNAH6 |
| 173 | Dynein heavy chain 7, axonemal | DNAH7 |
| 174 | Dynein heavy chain 8, axonemal | DNAH8 |
| 175 | Dynein heavy chain 9, axonemal | DNAH9 |
| 176 | Dynein heavy chain domain-containing protein 1 | DNHD1 |
| 177 | Dysferlin | DYSF |
| 178 | Dystonin | DST |
| 179 | E3 ISG15--protein ligase HERC5 | HERC5 |
| 180 | E3 ubiquitin-protein ligase HECTD1 | HECTD1 |
| 181 | E3 ubiquitin-protein ligase HERC2 | HERC2 |
| 182 | E3 ubiquitin-protein ligase HUWE1 | HUWE1 |
| 183 | E3 ubiquitin-protein ligase MYCBP2 | MYCBP2 |
| 184 | E3 ubiquitin-protein ligase UBR4 | UBR4 |
| 185 | E3 ubiquitin-protein ligase UBR5 | UBR5 |
| 186 | Early endosome antigen 1 | EEA1 |
| 187 | EF-hand calcium-binding domain-containing protein 6 | EFCAB6 |
| 188 | EH domain-binding protein 1 | EHBP1 |
| 189 | ELKS/Rab6-interacting/CAST family member 1 | ERC1 |
| 190 | Elongation factor 1-alpha 1 | EEF1A1 |
| 191 | Elongation factor 1-alpha 2 | EEF1A2 |
| 192 | Elongation factor 1-beta | EEF1B2 |
| 193 | Elongation factor 1-delta | EEF1D |
| 194 | Elongation factor 1-gamma | EEF1G |
| 195 | Elongation factor 2 | EEF2 |
| 196 | Endogenous retrovirus group K member 10 Pol protein;Reverse transcriptase;Ribonuclease H;Integrase;Endogenous retrovirus group K member 24 Gag polyprotein | ERVK-10;ERVK-24 |
| 197 | Endoplasmin | HSP90B1 |
| 198 | Epiplakin | EPPK1 |
| 199 | Eukaryotic initiation factor 4A-I;Eukaryotic initiation factor 4A-II;Eukaryotic initiation factor 4A-II, N-terminally processed | EIF4A1;EIF4A2 |
| 200 | Eukaryotic peptide chain release factor GTP-binding subunit ERF3A | GSPT1 |
| 201 | Eukaryotic translation initiation factor 3 subunit A | EIF3A |
| 202 | Eukaryotic translation initiation factor 3 subunit M | EIF3M |
| 203 | Eukaryotic translation initiation factor 5A-1;Eukaryotic translation initiation factor 5A-1-like | EIF5A;EIF5AL1 |
| 204 | Exportin-1 | XPO1 |
| 205 | Exportin-2 | CSE1L |
| 206 | Exportin-5 | XPO5 |
| 207 | Extracellular calcium-sensing receptor | CASR |
| 208 | Ezrin | EZR |
| 209 | F-actin-capping protein subunit beta | CAPZB |
| 210 | Farnesyl pyrophosphate synthase | FDPS |
| 211 | Fas-binding factor 1 | FBF1 |
| 212 | Fatty acid synthase;[Acyl-carrier-protein] S-acetyltransferase;[Acyl-carrier-protein] S-malonyltransferase;3-oxoacyl-[acyl-carrier-protein] synthase;3-oxoacyl-[acyl-carrier-protein] reductase;3-hydroxyacyl-[acyl-carrier-protein] dehydratase;Enoyl-[acyl-carrier-protein] reductase;Oleoyl-[acyl-carrier-protein] hydrolase | FASN |
| 213 | Fer-1-like protein 4 | FER1L4 |
| 214 | FERM and PDZ domain-containing protein 1 | FRMPD1 |
| 215 | FERM, RhoGEF and pleckstrin domain-containing protein 2 | FARP2 |
| 216 | Fibrillin-2 | FBN2 |
| 217 | Fibroblast growth factor receptor 2 | FGFR2 |
| 218 | Fibrocystin | PKHD1 |
| 219 | Fibrosin-1-like protein | FBRSL1 |
| 220 | Fibrous sheath-interacting protein 2 | FSIP2 |
| 221 | Filamin-A | FLNA |
| 222 | Filamin-B | FLNB |
| 223 | Filamin-C | FLNC |
| 224 | Flotillin-2 | FLOT2 |
| 225 | Forkhead-associated domain-containing protein 1 | FHAD1 |
| 226 | Formin-1 | FMN1 |
| 227 | FRAS1-related extracellular matrix protein 2 | FREM2 |
| 228 | Frizzled-3 | FZD3 |
| 229 | Fructose-bisphosphate aldolase A | ALDOA |
| 230 | G-protein coupled receptor 98 | GPR98 |
| 231 | G-protein coupled receptor family C group 6 member A | GPRC6A |
| 232 | Galactose-3-O-sulfotransferase 3 | GAL3ST3 |
| 233 | Galectin-3 | LGALS3 |
| 234 | General transcription factor II-I repeat domain-containing protein 1 | GTF2IRD1 |
| 235 | Girdin | CCDC88A |
| 236 | Glucose-6-phosphate isomerase | GPI |
| 237 | Glutamate dehydrogenase 1, mitochondrial | GLUD1 |
| 238 | Glutamine--fructose-6-phosphate aminotransferase [isomerizing] 1 | GFPT1 |
| 239 | Glutamyl-tRNA(Gln) amidotransferase subunit B, mitochondrial | GATB |
| 240 | Glutaredoxin-3 | GLRX3 |
| 241 | Glutathione reductase, mitochondrial | GSR |
| 242 | Glutathione S-transferase omega-1 | GSTO1 |
| 243 | Glutathione S-transferase P | GSTP1 |
| 244 | Glyceraldehyde-3-phosphate dehydrogenase | GAPDH |
| 245 | Glycine--tRNA ligase | GARS |
| 246 | Glycosyltransferase-like protein LARGE1;Xylosyltransferase LARGE;Beta-1,3-glucuronyltransferase LARGE | LARGE |
| 247 | Glypican-4;Secreted glypican-4 | GPC4 |
| 248 | Golgi integral membrane protein 4 | GOLIM4 |
| 249 | Golgin subfamily A member 4 | GOLGA4 |
| 250 | Golgin subfamily A member 8B;Golgin subfamily A member 8A | GOLGA8B;GOLGA8A |
| 251 | Golgin subfamily B member 1 | GOLGB1 |
| 252 | GTP-binding nuclear protein Ran | RAN |
| 253 | Guanine nucleotide exchange factor for Rab-3A | RAB3IL1 |
| 254 | Guanine nucleotide-binding protein subunit beta-2-like 1;Guanine nucleotide-binding protein subunit beta-2-like 1, N-terminally processed | GNB2L1 |
| 255 | HEAT repeat-containing protein 1;HEAT repeat-containing protein 1, N-terminally processed | HEATR1 |
| 256 | Heat shock 70 kDa protein 1B;Heat shock 70 kDa protein 1A | HSPA1B;HSPA1A |
| 257 | Heat shock 70 kDa protein 4 | HSPA4 |
| 258 | Heat shock 70 kDa protein 6 | HSPA6 |
| 259 | Heat shock cognate 71 kDa protein | HSPA8 |
| 260 | Heat shock protein 105 kDa | HSPH1 |
| 261 | Heat shock protein HSP 90-alpha | HSP90AA1 |
| 262 | Heat shock protein HSP 90-beta | HSP90AB1 |
| 263 | Helicase SRCAP | SRCAP |
| 264 | Helicase with zinc finger domain 2 | HELZ2 |
| 265 | Hephaestin | HEPH |
| 266 | Hermansky-Pudlak syndrome 3 protein | HPS3 |
| 267 | Heterogeneous nuclear ribonucleoprotein K | HNRNPK |
| 268 | Hexokinase-1 | HK1 |
| 269 | High affinity cAMP-specific and IBMX-insensitive 3,5-cyclic phosphodiesterase 8A | PDE8A |
| 270 | High mobility group protein B1;Putative high mobility group protein B1-like 1 | HMGB1;HMGB1P1 |
| 271 | Histidine triad nucleotide-binding protein 1 | HINT1 |
| 272 | Histone acetyltransferase KAT6A | KAT6A |
| 273 | Histone-lysine N-methyltransferase 2A;MLL cleavage product N320;MLL cleavage product C180 | KMT2A |
| 274 | Histone-lysine N-methyltransferase 2B | KMT2B |
| 275 | Histone-lysine N-methyltransferase 2D | KMT2D |
| 276 | Histone-lysine N-methyltransferase ASH1L | ASH1L |
| 277 | Histone-lysine N-methyltransferase SUV420H1 | SUV420H1 |
| 278 | Homeobox protein cut-like 2 | CUX2 |
| 279 | Hsc70-interacting protein;Putative protein FAM10A4;Putative protein FAM10A5 | ST13;ST13P4;ST13P5 |
| 280 | Huntingtin | HTT |
| 281 | Hydrocephalus-inducing protein homolog | HYDIN |
| 282 | Immunoglobulin superfamily member 1 | IGSF1 |
| 283 | Immunoglobulin superfamily member 10 | IGSF10 |
| 284 | Importin subunit alpha-1 | KPNA2 |
| 285 | Importin subunit beta-1 | KPNB1 |
| 286 | Importin-5 | IPO5 |
| 287 | Importin-7 | IPO7 |
| 288 | Importin-7 | IPO7 |
| 289 | Inactive phospholipase C-like protein 1 | PLCL1 |
| 290 | Inhibitor of Bruton tyrosine kinase | IBTK |
| 291 | Inner nuclear membrane protein Man1 | LEMD3 |
| 292 | Inorganic pyrophosphatase | PPA1 |
| 293 | Inositol 1,4,5-trisphosphate receptor type 2 | ITPR2 |
| 294 | Inositol hexakisphosphate and diphosphoinositol-pentakisphosphate kinase 2 | PPIP5K2 |
| 295 | Insulin receptor-related protein;Insulin receptor-related protein alpha chain;Insulin receptor-related protein beta chain | INSRR |
| 296 | Insulin-degrading enzyme | IDE |
| 297 | Insulinoma-associated protein 2 | INSM2 |
| 298 | Integrator complex subunit 1 | INTS1 |
| 299 | Integrin alpha-L | ITGAL |
| 300 | Integrin beta-2 | ITGB2 |
| 301 | Integrin beta-4 | ITGB4 |
| 302 | Interleukin-1 receptor-associated kinase-like 2 | IRAK2 |
| 303 | Interleukin-13 receptor subunit alpha-2 | IL13RA2 |
| 304 | Intraflagellar transport protein 172 homolog | IFT172 |
| 305 | Intron-binding protein aquarius | AQR |
| 306 | Isocitrate dehydrogenase [NADP] cytoplasmic | IDH1 |
| 307 | KAT8 regulatory NSL complex subunit 1 | KANSL1 |
| 308 | Keratin, type I cytoskeletal 18 | KRT18 |
| 309 | Kinectin | KTN1 |
| 310 | Kinesin-like protein KIF14 | KIF14 |
| 311 | Kinesin-like protein KIF15 | KIF15 |
| 312 | Kinesin-like protein KIF1C | KIF1C |
| 313 | Kinesin-like protein KIF21A | KIF21A |
| 314 | Kinetochore-associated protein 1 | KNTC1 |
| 315 | Krueppel-related zinc finger protein 1 | HKR1 |
| 316 | L-lactate dehydrogenase A chain | LDHA |
| 317 | L-lactate dehydrogenase B chain | LDHB |
| 318 | L-lactate dehydrogenase C chain | LDHC |
| 319 | Laminin subunit alpha-2 | LAMA2 |
| 320 | Laminin subunit alpha-5 | LAMA5 |
| 321 | Laminin subunit beta-1 | LAMB1 |
| 322 | Laminin subunit beta-2 | LAMB2 |
| 323 | Laminin subunit beta-4 | LAMB4 |
| 324 | Laminin subunit gamma-1 | LAMC1 |
| 325 | Leptin receptor | LEPR |
| 326 | Leucine-rich repeat and IQ domain-containing protein 3 | LRRIQ3 |
| 327 | Liprin-alpha-1 | PPFIA1 |
| 328 | Liprin-alpha-3 | PPFIA3 |
| 329 | Little elongation complex subunit 1 | ICE1 |
| 330 | Low-density lipoprotein receptor-related protein 8 | LRP8 |
| 331 | Lupus La protein | SSB |
| 332 | Lysine-specific demethylase 2B | KDM2B |
| 333 | Lysine-specific demethylase 3B | KDM3B |
| 334 | Lysosomal-trafficking regulator | LYST |
| 335 | M-phase phosphoprotein 8 | MPHOSPH8 |
| 336 | Macrophage migration inhibitory factor | MIF |
| 337 | Maestro heat-like repeat-containing protein family member 2A | MROH2A |
| 338 | Malate dehydrogenase, cytoplasmic | MDH1 |
| 339 | Malate dehydrogenase, mitochondrial | MDH2 |
| 340 | Maltase-glucoamylase, intestinal;Maltase;Glucoamylase | MGAM |
| 341 | MARVEL domain-containing protein 2 | MARVELD2 |
| 342 | Membrane-associated guanylate kinase, WW and PDZ domain-containing protein 2 | MAGI2 |
| 343 | Methionine synthase reductase | MTRR |
| 344 | Microtubule-actin cross-linking factor 1, isoforms 1/2/3/5 | MACF1 |
| 345 | Microtubule-associated protein 1B;MAP1B heavy chain;MAP1 light chain LC1 | MAP1B |
| 346 | Microtubule-associated serine/threonine-protein kinase 4 | MAST4 |
| 347 | Microtubule-associated tumor suppressor candidate 2 | MTUS2 |
| 348 | Midasin | MDN1 |
| 349 | Mimecan | OGN |
| 350 | Mitochondrial dynamics protein MID51 | MIEF1 |
| 351 | Mitogen-activated protein kinase kinase kinase 10 | MAP3K10 |
| 352 | MORC family CW-type zinc finger protein 1 | MORC1 |
| 353 | Msx2-interacting protein | SPEN |
| 354 | Mucin-2 | MUC2 |
| 355 | Mucin-5B | MUC5B |
| 356 | Multifunctional protein ADE2;Phosphoribosylaminoimidazole-succinocarboxamide synthase;Phosphoribosylaminoimidazole carboxylase | PAICS |
| 357 | Multimerin-1;Platelet glycoprotein Ia*;155 kDa platelet multimerin | MMRN1 |
| 358 | Myelin transcription factor 1 | MYT1 |
| 359 | Myomegalin | PDE4DIP |
| 360 | Myomesin-1 | MYOM1 |
| 361 | Myosin light polypeptide 6 | MYL6 |
| 362 | Myosin regulatory light chain 12B;Myosin regulatory light chain 12A | MYL12B;MYL12A |
| 363 | Myosin-1;Myosin-4 | MYH1;MYH4 |
| 364 | Myosin-10 | MYH10 |
| 365 | Myosin-11 | MYH11 |
| 366 | Myosin-13;Myosin-8 | MYH13;MYH8 |
| 367 | Myosin-15 | MYH15 |
| 368 | Myosin-6 | MYH6 |
| 369 | Myosin-7B | MYH7B |
| 370 | Myosin-9 | MYH9 |
| 371 | Myosin-binding protein C, cardiac-type | MYBPC3 |
| 372 | Myotubularin-related protein 12 | MTMR12 |
| 373 | Myotubularin-related protein 13 | SBF2 |
| 374 | Myotubularin-related protein 5 | SBF1 |
| 375 | NACHT, LRR and PYD domains-containing protein 14 | NLRP14 |
| 376 | NADP-dependent malic enzyme | ME1 |
| 377 | Nascent polypeptide-associated complex subunit alpha, muscle-specific form | NACA |
| 378 | Nck-associated protein 5 | NCKAP5 |
| 379 | Nebulette | NEBL |
| 380 | Nebulin | NEB |
| 381 | Nephrocystin-4 | NPHP4 |
| 382 | Nesprin-1 | SYNE1 |
| 383 | Nesprin-2 | SYNE2 |
| 384 | Nesprin-3 | SYNE3 |
| 385 | Netrin-1 | NTN1 |
| 386 | Neurobeachin-like protein 1 | NBEAL1 |
| 387 | Neuroblast differentiation-associated protein AHNAK | AHNAK |
| 388 | Neurofilament medium polypeptide | NEFM |
| 389 | Neutral alpha-glucosidase AB | GANAB |
| 390 | NFX1-type zinc finger-containing protein 1 | ZNFX1 |
| 391 | Nicotinate-nucleotide pyrophosphorylase [carboxylating] | QPRT |
| 392 | Non-receptor tyrosine-protein kinase TYK2 | TYK2 |
| 393 | Nuclear autoantigenic sperm protein | NASP |
| 394 | Nuclear mitotic apparatus protein 1 | NUMA1 |
| 395 | Nuclear pore complex-interacting protein family member B5;Nuclear pore complex-interacting protein family member B3;Nuclear pore complex-interacting protein family member B4;Putative NPIP-like protein LOC613037;Nuclear pore complex-interacting protein family member B11 | NPIPB5;NPIPB3;NPIPB4;NPIPB11 |
| 396 | Nuclear receptor corepressor 1 | NCOR1 |
| 397 | Nucleolar pre-ribosomal-associated protein 1 | URB1 |
| 398 | Nucleolar protein 8 | NOL8 |
| 399 | Nucleolar transcription factor 1 | UBTF |
| 400 | Nucleoside diphosphate kinase A | NME1 |
| 401 | Nucleoside diphosphate kinase B;Putative nucleoside diphosphate kinase | NME2;NME2P1 |
| 402 | Nucleosome assembly protein 1-like 1 | NAP1L1 |
| 403 | Nucleosome-remodeling factor subunit BPTF | BPTF |
| 404 | Obscurin | OBSCN |
| 405 | Obscurin-like protein 1 | OBSL1 |
| 406 | Paired amphipathic helix protein Sin3a | SIN3A |
| 407 | Papilin | PAPLN |
| 408 | Paralemmin-2 | PALM2-AKAP2;PALM2 |
| 409 | Patatin-like phospholipase domain-containing protein 7 | PNPLA7 |
| 410 | Patched domain-containing protein 2 | PTCHD2 |
| 411 | PDZ and LIM domain protein 1 | PDLIM1 |
| 412 | PDZ domain-containing protein 2;Processed PDZ domain-containing protein 2 | PDZD2 |
| 413 | Peptidyl-glycine alpha-amidating monooxygenase;Peptidylglycine alpha-hydroxylating monooxygenase;Peptidyl-alpha-hydroxyglycine alpha-amidating lyase | PAM |
| 414 | Peptidyl-prolyl cis-trans isomerase A;Peptidyl-prolyl cis-trans isomerase A, N-terminally processed | PPIA |
| 415 | Periaxin | PRX |
| 416 | Pericentrin | PCNT |
| 417 | Peripheral plasma membrane protein CASK | CASK |
| 418 | Peripheral-type benzodiazepine receptor-associated protein 1 | BZRAP1 |
| 419 | Peroxidasin-like protein | PXDNL |
| 420 | Peroxiredoxin-1 | PRDX1 |
| 421 | Peroxiredoxin-2 | PRDX2 |
| 422 | Peroxiredoxin-5, mitochondrial | PRDX5 |
| 423 | Peroxiredoxin-6 | PRDX6 |
| 424 | Peroxisome proliferator-activated receptor gamma coactivator-related protein 1 | PPRC1 |
| 425 | Phosphatidylethanolamine-binding protein 1;Hippocampal cholinergic neurostimulating peptide | PEBP1 |
| 426 | Phosphatidylinositol 3,4,5-trisphosphate-dependent Rac exchanger 2 protein | PREX2 |
| 427 | Phosphoglycerate kinase 1 | PGK1 |
| 428 | Phosphoglycerate mutase 1 | PGAM1 |
| 429 | Phospholipid-transporting ATPase IB | ATP8A2 |
| 430 | Phosphoribosyl pyrophosphate synthase-associated protein 1 | PRPSAP1 |
| 431 | Piwi-like protein 1 | PIWIL1 |
| 432 | Plastin-2 | LCP1 |
| 433 | Plastin-3 | PLS3 |
| 434 | Platelet-activating factor acetylhydrolase IB subunit beta | PAFAH1B2 |
| 435 | Pleckstrin homology domain-containing family A member 6 | PLEKHA6 |
| 436 | Pleckstrin homology domain-containing family H member 1 | PLEKHH1 |
| 437 | Plectin | PLEC |
| 438 | Plexin-A3 | PLXNA3 |
| 439 | Polyamine-modulated factor 1-binding protein 1 | PMFBP1 |
| 440 | Polycystic kidney disease and receptor for egg jelly-related protein | PKDREJ |
| 441 | Polycystic kidney disease protein 1-like 1 | PKD1L1 |
| 442 | PR domain-containing protein 11 | PRDM11 |
| 443 | Pre-mRNA-processing-splicing factor 8 | PRPF8 |
| 444 | Pro-epidermal growth factor;Epidermal growth factor | EGF |
| 445 | Pro-neuregulin-2, membrane-bound isoform;Neuregulin-2 | NRG2 |
| 446 | Probable ATP-dependent RNA helicase DDX60-like | DDX60L |
| 447 | Probable cation-transporting ATPase 13A5 | ATP13A5 |
| 448 | Probable E3 ubiquitin-protein ligase HERC1 | HERC1 |
| 449 | Probable E3 ubiquitin-protein ligase HERC3 | HERC3 |
| 450 | Probable E3 ubiquitin-protein ligase TRIML2 | TRIML2 |
| 451 | Probable global transcription activator SNF2L1 | SMARCA1 |
| 452 | Probable helicase with zinc finger domain | HELZ |
| 453 | Probable JmjC domain-containing histone demethylation protein 2C | JMJD1C |
| 454 | Probable phospholipid-transporting ATPase IIB | ATP9B |
| 455 | Procollagen-lysine,2-oxoglutarate 5-dioxygenase 2 | PLOD2 |
| 456 | Profilin-1 | PFN1 |
| 457 | Proliferating cell nuclear antigen | PCNA |
| 458 | Proline-rich protein 14 | PRR14 |
| 459 | Prolyl endopeptidase-like | PREPL |
| 460 | Prosaposin;Saposin-A;Saposin-B-Val;Saposin-B;Saposin-C;Saposin-D | PSAP |
| 461 | Prostaglandin E synthase 3 | PTGES3 |
| 462 | Proteasome activator complex subunit 1 | PSME1 |
| 463 | Protein bassoon | BSN |
| 464 | Protein canopy homolog 2 | CNPY2 |
| 465 | Protein CASC5 | CASC5 |
| 466 | Protein DBF4 homolog A | DBF4 |
| 467 | Protein deglycase DJ-1 | PARK7 |
| 468 | Protein diaphanous homolog 1 | DIAPH1 |
| 469 | Protein diaphanous homolog 2 | DIAPH2 |
| 470 | Protein disulfide-isomerase | P4HB |
| 471 | Protein disulfide-isomerase A3 | PDIA3 |
| 472 | Protein disulfide-isomerase A4 | PDIA4 |
| 473 | Protein disulfide-isomerase A6 | PDIA6 |
| 474 | Protein eyes shut homolog | EYS |
| 475 | Protein FAM171A2 | FAM171A2 |
| 476 | Protein FAM184B | FAM184B |
| 477 | Protein FAM208B | FAM208B |
| 478 | Protein kinase C epsilon type | PRKCE |
| 479 | Protein NRDE2 homolog | NRDE2 |
| 480 | Protein PAT1 homolog 2 | PATL2 |
| 481 | Protein phosphatase 1 regulatory subunit 14B | PPP1R14B |
| 482 | Protein phosphatase 1 regulatory subunit 37 | PPP1R37 |
| 483 | Protein piccolo | PCLO |
| 484 | Protein polybromo-1 | PBRM1 |
| 485 | Protein PRRC2B | PRRC2B |
| 486 | Protein PTHB1 | BBS9 |
| 487 | Protein RRP5 homolog | PDCD11 |
| 488 | Protein S100-A11;Protein S100-A11, N-terminally processed | S100A11 |
| 489 | Protein S100-A6 | S100A6 |
| 490 | Protein SET;Protein SETSIP | SET;SETSIP |
| 491 | Protein SFI1 homolog | SFI1 |
| 492 | Protein SGT1 | ECD |
| 493 | Protein Shroom2 | SHROOM2 |
| 494 | Protein SMG7 | SMG7 |
| 495 | Protein SOGA1;N-terminal form;C-terminal 80 kDa form | SOGA1 |
| 496 | Protein SON | SON |
| 497 | Protein strawberry notch homolog 2 | SBNO2 |
| 498 | Protein SZT2 | SZT2 |
| 499 | Protein TESPA1 | TESPA1 |
| 500 | Protein transport protein Sec31A | SEC31A |
| 501 | Protein virilizer homolog | KIAA1429 |
| 502 | Prothymosin alpha;Prothymosin alpha, N-terminally processed;Thymosin alpha-1 | PTMA |
| 503 | Proto-oncogene vav | VAV1 |
| 504 | Protocadherin Fat 4 | FAT4 |
| 505 | Puromycin-sensitive aminopeptidase | NPEPPS |
| 506 | Putative beta-lactamase-like 1 | LACTBL1 |
| 507 | Putative heat shock protein HSP 90-alpha A4 | HSP90AA4P |
| 508 | Putative heat shock protein HSP 90-beta 2 | HSP90AB2P |
| 509 | Putative NPIP-like protein LOC729978 |  |
| 510 | Putative RNA-binding protein Luc7-like 1 | LUC7L |
| 511 | Putative RNA-binding protein Luc7-like 2 | C7orf55-LUC7L2;LUC7L2 |
| 512 | Putative zinc finger protein 833 | ZNF833P |
| 513 | Pyridoxal kinase | PDXK |
| 514 | Pyruvate kinase PKM | PKM |
| 515 | R-spondin-2 | RSPO2 |
| 516 | Rab GDP dissociation inhibitor beta | GDI2 |
| 517 | RAC-alpha serine/threonine-protein kinase | AKT1 |
| 518 | Ras GTPase-activating-like protein IQGAP1 | IQGAP1 |
| 519 | Ras GTPase-activating-like protein IQGAP2 | IQGAP2 |
| 520 | RB1-inducible coiled-coil protein 1 | RB1CC1 |
| 521 | Receptor tyrosine-protein kinase erbB-2 | ERBB2 |
| 522 | Receptor-type tyrosine-protein phosphatase gamma | PTPRG |
| 523 | Receptor-type tyrosine-protein phosphatase kappa | PTPRK |
| 524 | Regulator of nonsense transcripts 2 | UPF2 |
| 525 | Remodeling and spacing factor 1 | RSF1 |
| 526 | Reticulon-4 | RTN4 |
| 527 | Retinal dehydrogenase 1 | ALDH1A1 |
| 528 | Retinoic acid receptor RXR-alpha | RXRA |
| 529 | Rho GTPase-activating protein 21 | ARHGAP21 |
| 530 | Rho GTPase-activating protein 32 | ARHGAP32 |
| 531 | Rho GTPase-activating protein 33 | ARHGAP33 |
| 532 | Rho GTPase-activating protein 35 | ARHGAP35 |
| 533 | Rho guanine nucleotide exchange factor 38 | ARHGEF38 |
| 534 | Rho-associated protein kinase 2 | ROCK2 |
| 535 | Rhophilin-2 | RHPN2 |
| 536 | RIMS-binding protein 3B;RIMS-binding protein 3C;RIMS-binding protein 3A | RIMBP3B;RIMBP3C;RIMBP3 |
| 537 | RING finger protein unkempt homolog | UNK |
| 538 | Ribonuclease inhibitor | RNH1 |
| 539 | RNA polymerase II transcription factor SIII subunit A3;RNA polymerase II transcription factor SIII subunit A3-like-1;RNA polymerase II transcription factor SIII subunit A3-like-2 | LOC101930165;TCEB3C;TCEB3CL;TCEB3CL2 |
| 540 | RNA-binding protein 12B | RBM12B |
| 541 | Rod cGMP-specific 3,5-cyclic phosphodiesterase subunit alpha | PDE6A |
| 542 | Ryanodine receptor 2 | RYR2 |
| 543 | Ryanodine receptor 3 | RYR3 |
| 544 | S1 RNA-binding domain-containing protein 1 | SRBD1 |
| 545 | Scaffold attachment factor B1 | SAFB |
| 546 | Schlafen family member 11 | SLFN11 |
| 547 | Sciellin | SCEL |
| 548 | Sentrin-specific protease 7 | SENP7 |
| 549 | Serine protease inhibitor Kazal-type 5;Hemofiltrate peptide HF6478;Hemofiltrate peptide HF7665 | SPINK5 |
| 550 | Serine/arginine repetitive matrix protein 2 | SRRM2 |
| 551 | Serine/threonine-protein kinase 25 | STK25 |
| 552 | Serine/threonine-protein kinase DCLK1 | DCLK1 |
| 553 | Serine/threonine-protein kinase N1 | PKN1 |
| 554 | Serine/threonine-protein kinase Nek1 | NEK1 |
| 555 | Serine/threonine-protein kinase Nek5 | NEK5 |
| 556 | Serine/threonine-protein kinase PLK3 | PLK3 |
| 557 | Serine/threonine-protein kinase SMG1 | SMG1 |
| 558 | Serine/threonine-protein phosphatase 2A 65 kDa regulatory subunit A alpha isoform | PPP2R1A |
| 559 | Serpin H1 | SERPINH1 |
| 560 | SET-binding protein | SETBP1 |
| 561 | SH3 and multiple ankyrin repeat domains protein 1 | SHANK1 |
| 562 | SH3 domain-containing kinase-binding protein 1 | SH3KBP1 |
| 563 | SLIT-ROBO Rho GTPase-activating protein 2 | SRGAP2 |
| 564 | Slit homolog 3 protein | SLIT3 |
| 565 | Sodium channel protein type 10 subunit alpha | SCN10A |
| 566 | Sodium channel protein type 11 subunit alpha | SCN11A |
| 567 | Soluble scavenger receptor cysteine-rich domain-containing protein SSC5D | SSC5D |
| 568 | Sorbin and SH3 domain-containing protein 2 | SORBS2 |
| 569 | Sortilin-related receptor | SORL1 |
| 570 | Sorting nexin-14 | SNX14 |
| 571 | Sorting nexin-2 | SNX2 |
| 572 | Spectrin alpha chain, non-erythrocytic 1 | SPTAN1 |
| 573 | Spectrin beta chain, non-erythrocytic 1 | SPTBN1 |
| 574 | Spectrin beta chain, non-erythrocytic 4 | SPTBN4 |
| 575 | Spermatogenesis-associated protein 5-like protein 1 | SPATA5L1 |
| 576 | Spermatogenesis-associated protein 6 | SPATA6 |
| 577 | Staphylococcal nuclease domain-containing protein 1 | SND1 |
| 578 | Starch-binding domain-containing protein 1 | STBD1 |
| 579 | Stathmin | STMN1 |
| 580 | Sterile alpha motif domain-containing protein 15 | SAMD15 |
| 581 | Sterile alpha motif domain-containing protein 9 | SAMD9 |
| 582 | Stress-70 protein, mitochondrial | HSPA9 |
| 583 | Stress-induced-phosphoprotein 1 | STIP1 |
| 584 | Striated muscle preferentially expressed protein kinase | SPEG |
| 585 | Structural maintenance of chromosomes flexible hinge domain-containing protein 1 | SMCHD1 |
| 586 | Structural maintenance of chromosomes protein 1A | SMC1A |
| 587 | Structural maintenance of chromosomes protein 1B | SMC1B |
| 588 | Structural maintenance of chromosomes protein 2 | SMC2 |
| 589 | Structural maintenance of chromosomes protein 4 | SMC4 |
| 590 | Sushi, von Willebrand factor type A, EGF and pentraxin domain-containing protein 1 | SVEP1 |
| 591 | Synaptonemal complex protein 1 | SYCP1 |
| 592 | Synaptotagmin-like protein 5 | SYTL5 |
| 593 | T-complex protein 1 subunit alpha | TCP1 |
| 594 | T-complex protein 1 subunit epsilon | CCT5 |
| 595 | T-complex protein 1 subunit eta | CCT7 |
| 596 | Talin-1 | TLN1 |
| 597 | Telomerase protein component 1 | TEP1 |
| 598 | Teneurin-1;Ten-1 intracellular domain;Teneurin C-terminal-associated peptide | TENM1 |
| 599 | Teneurin-4 | TENM4 |
| 600 | Terminal uridylyltransferase 7 | ZCCHC6 |
| 601 | TFIIH basal transcription factor complex helicase XPB subunit | ERCC3 |
| 602 | Thioredoxin | TXN |
| 603 | Thioredoxin reductase 1, cytoplasmic | TXNRD1 |
| 604 | Threonine--tRNA ligase, cytoplasmic | TARS |
| 605 | Thyroglobulin | TG |
| 606 | Thyroid hormone receptor beta | THRB |
| 607 | Thyroid receptor-interacting protein 11 | TRIP11 |
| 608 | Titin | TTN |
| 609 | Transaldolase | TALDO1 |
| 610 | Transcription factor TFIIIB component B homolog | BDP1 |
| 611 | Transcription initiation factor TFIID subunit 1 | TAF1 |
| 612 | Transcription intermediary factor 1-beta | TRIM28 |
| 613 | Transferrin receptor protein 1;Transferrin receptor protein 1, serum form | TFRC |
| 614 | Transformation/transcription domain-associated protein | TRRAP |
| 615 | Transgelin | TAGLN |
| 616 | Transgelin-2 | TAGLN2 |
| 617 | Transient receptor potential cation channel subfamily M member 6 | TRPM6 |
| 618 | Transient receptor potential cation channel subfamily V member 1 | TRPV1 |
| 619 | Transitional endoplasmic reticulum ATPase | VCP |
| 620 | Transketolase | TKT |
| 621 | Translationally-controlled tumor protein | TPT1 |
| 622 | Transmembrane and TPR repeat-containing protein 3 | TMTC3 |
| 623 | Transmembrane protein 132D | TMEM132D |
| 624 | Treslin | TICRR |
| 625 | Triadin | TRDN |
| 626 | Trifunctional purine biosynthetic protein adenosine-3;Phosphoribosylamine--glycine ligase;Phosphoribosylformylglycinamidine cyclo-ligase;Phosphoribosylglycinamide formyltransferase | GART |
| 627 | Triosephosphate isomerase | TPI1 |
| 628 | Triple functional domain protein | TRIO |
| 629 | tRNA (adenine(58)-N(1))-methyltransferase, mitochondrial | TRMT61B |
| 630 | Tropomyosin alpha-3 chain | TPM3 |
| 631 | Tropomyosin alpha-4 chain;Tropomyosin alpha-1 chain | TPM4;TPM1 |
| 632 | Tubulin alpha-1B chain;Tubulin alpha-4A chain | TUBA1B;TUBA4A |
| 633 | Tubulin alpha-1C chain | TUBA1C |
| 634 | Tubulin beta chain | TUBB |
| 635 | Tubulin beta-2B chain;Tubulin beta-2A chain | TUBB2B;TUBB2A |
| 636 | Tubulin beta-4A chain | TUBB4A |
| 637 | Tubulin beta-4B chain | TUBB4B |
| 638 | Tubulin beta-6 chain | TUBB6 |
| 639 | Tubulin beta-8 chain | TUBB8 |
| 640 | Tudor domain-containing protein 15 | TDRD15 |
| 641 | Tudor domain-containing protein 6 | TDRD6 |
| 642 | Tumor protein D54 | TPD52L2 |
| 643 | Tyrosine-protein phosphatase non-receptor type 1 | PTPN1 |
| 644 | Tyrosine-protein phosphatase non-receptor type 13 | PTPN13 |
| 645 | U2 small nuclear ribonucleoprotein auxiliary factor 35 kDa subunit-related protein 2 | ZRSR2 |
| 646 | Ubiquitin carboxyl-terminal hydrolase 24 | USP24 |
| 647 | Ubiquitin carboxyl-terminal hydrolase 34 | USP34 |
| 648 | Ubiquitin carboxyl-terminal hydrolase 5 | USP5 |
| 649 | Ubiquitin carboxyl-terminal hydrolase isozyme L1 | UCHL1 |
| 650 | Ubiquitin-60S ribosomal protein L40;Ubiquitin;60S ribosomal protein L40;Ubiquitin-40S ribosomal protein S27a;Ubiquitin;40S ribosomal protein S27a;Polyubiquitin-B;Ubiquitin;Polyubiquitin-C;Ubiquitin | UBA52;RPS27A;UBB;UBC |
| 651 | Ubiquitin-conjugating enzyme E2 L3 | UBE2L3;hCG_1789329 |
| 652 | Ubiquitin-conjugating enzyme E2 variant 1 | TMEM189-UBE2V1;UBE2V1 |
| 653 | Ubiquitin-fold modifier 1 | UFM1 |
| 654 | Ubiquitin-like modifier-activating enzyme 1 | UBA1 |
| 655 | UDP-glucose 6-dehydrogenase | UGDH |
| 656 | Uncharacterized protein C10orf71 | C10orf71 |
| 657 | Uncharacterized protein C2orf16 | C2orf16 |
| 658 | Uncharacterized protein C6orf118 | C6orf118 |
| 659 | Uncharacterized protein KIAA0232 | KIAA0232 |
| 660 | Uncharacterized protein KIAA0556 | KIAA0556 |
| 661 | Uncharacterized protein KIAA1109 | KIAA1109 |
| 662 | Uncharacterized protein KIAA1551 | KIAA1551 |
| 663 | Unconventional myosin-Ic | MYO1C |
| 664 | Unconventional myosin-IXa | MYO9A |
| 665 | Unconventional myosin-Vb | MYO5B |
| 666 | Unconventional myosin-X | MYO10 |
| 667 | Unconventional myosin-XVIIIa | MYO18A |
| 668 | Unconventional myosin-XVIIIb | MYO18B |
| 669 | Usherin | USH2A |
| 670 | UTP--glucose-1-phosphate uridylyltransferase | UGP2 |
| 671 | UV excision repair protein RAD23 homolog B | RAD23B |
| 672 | Uveal autoantigen with coiled-coil domains and ankyrin repeats | UACA |
| 673 | Vacuolar protein sorting-associated protein 13A | VPS13A |
| 674 | Vacuolar protein sorting-associated protein 13C | VPS13C |
| 675 | Vacuolar protein sorting-associated protein 13D | VPS13D |
| 676 | Vacuolar protein sorting-associated protein 35 | VPS35 |
| 677 | Vascular endothelial growth factor receptor 1 | FLT1 |
| 678 | Villin-1 | VIL1 |
| 679 | Vinculin | VCL |
| 680 | Voltage-dependent P/Q-type calcium channel subunit alpha-1A | CACNA1A |
| 681 | VPS10 domain-containing receptor SorCS1 | SORCS1 |
| 682 | WD repeat-containing protein 35 | WDR35 |
| 683 | WD repeat-containing protein 87 | WDR87 |
| 684 | X-ray repair cross-complementing protein 5 | XRCC5 |
| 685 | X-ray repair cross-complementing protein 6 | XRCC6 |
| 686 | Xaa-Pro aminopeptidase 1 | XPNPEP1 |
| 687 | Xaa-Pro dipeptidase | PEPD |
| 688 | Xenotropic and polytropic retrovirus receptor 1 | XPR1 |
| 689 | Xin actin-binding repeat-containing protein 2 | XIRP2 |
| 690 | Zinc finger and BTB domain-containing protein 11 | ZBTB11 |
| 691 | Zinc finger CCCH domain-containing protein 13 | ZC3H13 |
| 692 | Zinc finger homeobox protein 4 | ZFHX4 |
| 693 | Zinc finger MYM-type protein 6 | ZMYM6 |
| 694 | Zinc finger protein 30 | ZNF30 |
| 695 | Zinc finger protein 311 | ZNF311 |
| 696 | Zinc finger protein 319 | ZNF319 |
| 697 | Zinc finger protein 445 | ZNF445 |
| 698 | Zinc finger protein 518A | ZNF518A |
| 699 | Zinc finger protein 623 | ZNF623 |
| 700 | Zinc finger protein 646 | ZNF646 |
| 701 | Zinc finger protein 668 | ZNF668 |
| 702 | Zinc finger protein 708 | ZNF708 |
| 703 | Zinc finger protein 777 | ZNF777 |
| 704 | Zinc finger protein 836 | ZNF836 |
| 705 | Zinc finger protein 841 | ZNF841 |
| 706 | Zinc finger protein 860 | ZNF860 |
| 707 | Zinc finger protein 99 | ZNF99 |
| 708 | Zinc finger protein castor homolog 1 | CASZ1 |
| 709 | Zinc finger protein neuro-d4 | DPF1 |
| 710 | Zinc finger protein with KRAB and SCAN domains 1 | ZKSCAN1 |
| 711 | Zinc finger transcription factor Trps1 | TRPS1 |
