# Supplementary material for: Analysis of the Antiproliferative Effect of Ankaferd Hemostat on Caco-2 Colon Cancer Cells via LC/MS Shotgun Proteomics Approach
Source: Biomed Res Int. 2019 May 21;2019:5268031. doi: 10.1155/2019/5268031 (PMC6556321; doi:10.1155/2019/5268031)
Supplement: Supplementary Materials — Table S1. In proteomics analysis of Caco-2 cells, 711 proteins were identified in control and Ankaferd (ABS) treated groups. The identified proteins with their names and gene symbols are presented in Table 1. Table S2. The list of down- and upregulated proteins under ABS stress with their gene symbols and fold changes (p<0.05) [file 5268031.f1.zip › 5268031.f1/Table S2. Supplementary Material description_BMRI_2767462.docx]

**Table S2. Supplementary Material description**

The list of down and up regulated proteins under ABS stress with their gene symbols and fold changes (p<0.05)

**Table S2.** Increased proteins with fold change in Log2 (p<0.05)

| **Gene Symbol** | **Protein name** | **Fold Change (Log2)** |
| --- | --- | --- |
| PDIA3 | Protein disulfide-isomerase A3 | 4.75 |
| RAD23B | UV excision repair protein RAD23 homolog B | 3.73 |
| PRDX5 | Peroxiredoxin-5, mitochondrial | 3.67 |
| IL13RA2 | Interleukin-13 receptor subunit alpha-2 | 3.58 |
| HSPA9 | Stress-70 protein, mitochondrial | 3.37 |
| ACTN1 | Alpha-actinin-1 | 3.33 |
| RYR3 | Ryanodine receptor 3 | 3.19 |
| FBF1 | Fas-binding factor 1 | 2.57 |
| PSME1 | Proteasome activator complex subunit 1 | 2.53 |
| PTMA | Prothymosin alpha;Prothymosin alpha, N-terminally processed;Thymosin alpha-1 | 2.23 |
| HSPD1 | 60 kDa heat shock protein, mitochondrial | 2.08 |
| HSPE1 | 10 kDa heat shock protein, mitochondrial | 2.01 |
| S100A6 | Protein S100-A6 | 1.95 |
| CNPY2 | Protein canopy homolog 2 | 1.83 |
| MIF | Macrophage migration inhibitory factor | 1.65 |
| HSP90B1 | Endoplasmin | 1.56 |
| CLTC | Clathrin heavy chain 1 | 1.40 |
| PPP1R14B | Protein phosphatase 1 regulatory subunit 14B | 1.18 |
| AGR2 | Anterior gradient protein 2 homolog | 1.15 |
| PDXK | Pyridoxal kinase | 1.09 |
| CAP1 | Adenylyl cyclase-associated protein 1 | 1.09 |
| PEBP1 | Phosphatidylethanolamine-binding protein 1;Hippocampal cholinergic neurostimulating peptide | 1.04 |
| YWHAB | 14-3-3 protein beta/alpha;14-3-3 protein beta/alpha, N-terminally processed | 1.00 |
| ARF1;ARF3 | ADP-ribosylation factor 1;ADP-ribosylation factor 3 | 0.99 |
| PRDX2 | Peroxiredoxin-2 | 0.97 |
| GANAB | Neutral alpha-glucosidase AB | 0.95 |
| PDIA6 | Protein disulfide-isomerase A6 | 0.93 |
| S100A11 | Protein S100-A11;Protein S100-A11, N-terminally processed | 0.89 |
| RPL5 | 60S ribosomal protein L5 | 0.84 |
| PAFAH1B2 | Platelet-activating factor acetylhydrolase IB subunit beta | 0.81 |
| NPEPPS | Puromycin-sensitive aminopeptidase | 0.80 |
| UGDH | UDP-glucose 6-dehydrogenase | 0.80 |
| GFPT1 | Glutamine--fructose-6-phosphate aminotransferase [isomerizing] 1 | 0.77 |
| HSPA1B;HSPA1A | Heat shock 70 kDa protein 1B;Heat shock 70 kDa protein 1A | 0.75 |
| ACTN4 | Alpha-actinin-4 | 0.75 |
| HSPA5 | 78 kDa glucose-regulated protein | 0.73 |
| EIF5A;EIF5AL1 | Eukaryotic translation initiation factor 5A-1;Eukaryotic translation initiation factor 5A-1-like | 0.71 |
| IDH1 | Isocitrate dehydrogenase [NADP] cytoplasmic | 0.70 |
| UCHL1 | Ubiquitin carboxyl-terminal hydrolase isozyme L1 | 0.69 |
| P4HB | Protein disulfide-isomerase | 0.69 |
| PFKP | ATP-dependent 6-phosphofructokinase, platelet type | 0.68 |
| GOT1 | Aspartate aminotransferase, cytoplasmic | 0.65 |
| LDHB | L-lactate dehydrogenase B chain | 0.64 |
| ALDH1A1 | Retinal dehydrogenase 1 | 0.63 |
| SET;SETSIP | Protein SET;Protein SETSIP | 0.63 |
| GARS | Glycine--tRNA ligase | 0.61 |
| TXN | Thioredoxin | 0.58 |
| UBE2L3;hCG_1789329 | Ubiquitin-conjugating enzyme E2 L3 | 0.57 |
| CDK1 | Cyclin-dependent kinase 1 | 0.55 |
| PHGDH | D-3-phosphoglycerate dehydrogenase | 0.54 |
| AARS | Alanine--tRNA ligase, cytoplasmic | 0.54 |
| CALR | Calreticulin | 0.53 |
| PLS3 | Plastin-3 | 0.53 |
| ME1 | NADP-dependent malic enzyme | 0,53 |
| SSB | Lupus La protein | 0,53 |
| CCT7 | T-complex protein 1 subunit eta | 0.49 |
| PEPD | Xaa-Pro dipeptidase | 0.49 |
| ABRACL | Costars family protein ABRACL | 0.47 |
| ACTG1 | Actin, cytoplasmic 2;Actin, cytoplasmic 2, N-terminally processed | 0.47 |
| GPI | Glucose-6-phosphate isomerase | 0.45 |
| SERPINH1 | Serpin H1 | 0.44 |
| YWHAE | 14-3-3 protein epsilon | 0.44 |
| QPRT | Nicotinate-nucleotide pyrophosphorylase [carboxylating] | 0.41 |
| PTGES3 | Prostaglandin E synthase 3 | 0.39 |
| IPO7 | Importin-7 | 0.38 |
| PRDX1 | Peroxiredoxin-1 | 0.38 |
| APRT | Adenine phosphoribosyltransferase | 0.37 |
| NME1 | Nucleoside diphosphate kinase A | 0.37 |
| ANXA5 | Annexin A5 | 0.36 |
| GSTO1 | Glutathione S-transferase omega-1 | 0.34 |
| NASP | Nuclear autoantigenic sperm protein | 0.33 |
| HSPA4 | Heat shock 70 kDa protein 4 | 0.32 |
| GSTP1 | Glutathione S-transferase P | 0.32 |
| GAPDH | Glyceraldehyde-3-phosphate dehydrogenase | 0.31 |
| LDHA | L-lactate dehydrogenase A chain | 0.31 |
| PRDX6 | Peroxiredoxin-6 | 0.30 |
| PSMD13 | 26S proteasome non-ATPase regulatory subunit 13 | 0.29 |
| IQGAP1 | Ras GTPase-activating-like protein IQGAP1 | 0.27 |
| COPG1 | Coatomer subunit gamma-1 | 0.27 |
| TALDO1 | Transaldolase | 0.24 |
| CKB | Creatine kinase B-type | 0.22 |
| ACTR2 | Actin-related protein 2 | 0.22 |
| TKT | Transketolase | 0.22 |
| TUBA1C | Tubulin alpha-1C chain | 0.21 |
| ADSL | Adenylosuccinate lyase | 0.21 |
| STIP1 | Stress-induced-phosphoprotein 1 | 0.21 |
| CFL1 | Cofilin-1 | 0.21 |
| USP5 | Ubiquitin carboxyl-terminal hydrolase 5 | 0.18 |
| COPB1 | Coatomer subunit beta | 0.18 |
| CPNE1 | Copine-1 | 0.17 |
| ATIC | Bifunctional purine biosynthesis protein PURH;Phosphoribosylaminoimidazolecarboxamide formyltransferase;IMP cyclohydrolase | 0.16 |
| TAGLN2 | Transgelin-2 | 0.15 |
| XRCC5 | X-ray repair cross-complementing protein 5 | 0.14 |
| PFN1 | Profilin-1 | 0.14 |
| PGK1 | Phosphoglycerate kinase 1 | 0.13 |
| UBA1 | Ubiquitin-like modifier-activating enzyme 1 | 0.13 |
| ACTR3 | Actin-related protein 3 | 0.12 |
| CSE1L | Exportin-2 | 0.11 |
| ENO1 | Alpha-enolase | 0.09 |
| TUBA1B;TUBA4A | Tubulin alpha-1B chain;Tubulin alpha-4A chain | 0.06 |

**Table 2.** Decreased proteins with fold change in Log2 (p<0.05)

| **Gene Symbol** | **Protein name** | **Fold change (Log2)** |
| --- | --- | --- |
| TTN | Titin | -7.12 |
| BBS9 | Protein PTHB1 | -6.83 |
| RSPO2 | R-spondin-2 | -6.68 |
| PCLO | Protein piccolo | -5.47 |
| FAM208B | Protein FAM208B | -4.35 |
| RTN4 | Reticulon-4 | -4.17 |
| HNRNPK | Heterogeneous nuclear ribonucleoprotein K | -3.71 |
| RAB3IL1 | Guanine nucleotide exchange factor for Rab-3A | -3.30 |
| EEF1D | Elongation factor 1-delta | -3.26 |
| TRIM28 | Transcription intermediary factor 1-beta | -3.18 |
| CACNA1A | Voltage-dependent P/Q-type calcium channel subunit alpha-1A | -3.14 |
| TPD52L2 | Tumor protein D54 | -3.12 |
| UBTF | Nucleolar transcription factor 1 | -3.01 |
| CCDC93 | Coiled-coil domain-containing protein 93 | -2.72 |
| NEK5 | Serine/threonine-protein kinase Nek5 | -2.68 |
| ITPR2 | Inositol 1,4,5-trisphosphate receptor type 2 | -2.66 |
| TCP1 | T-complex protein 1 subunit alpha | -2.52 |
| KPNA2 | Importin subunit alpha-1 | -2.45 |
| SND1 | Staphylococcal nuclease domain-containing protein 1 | -2.23 |
| UFM1 | Ubiquitin-fold modifier 1 | -2.22 |
| CHMP4B | Charged multivesicular body protein 4b | -2.15 |
| CMYA5 | Cardiomyopathy-associated protein 5 | -2.14 |
| PDE4DIP | Myomegalin | -2.00 |
| EIF3M | Eukaryotic translation initiation factor 3 subunit M | -1.90 |
| EEF1G | Elongation factor 1-gamma | -1.84 |
| SNX2 | Sorting nexin-2 | -1.65 |
| MYO1C | Unconventional myosin-Ic | -1.63 |
| NACA | Nascent polypeptide-associated complex subunit alpha, muscle-specific form | -1.54 |
| EEF1B2 | Elongation factor 1-beta | -1.40 |
| TAGLN | Transgelin | -1.36 |
| PARK7 | Protein deglycase DJ-1 | -1.29 |
| PSMD1 | 26S proteasome non-ATPase regulatory subunit 1 | -1.18 |
| ANP32A | Acidic leucine-rich nuclear phosphoprotein 32 family member A | -1.03 |
| LGALS3 | Galectin-3 | -0.85 |
| CAPZB | F-actin-capping protein subunit beta | -0.80 |
| RPS12 | 40S ribosomal protein S12 | -0,77 |
| TFRC | Transferrin receptor protein 1;Transferrin receptor protein 1, serum form | -0.71 |
| YWHAG | 14-3-3 protein gamma;14-3-3 protein gamma, N-terminally processed | -0.70 |
| ARF4 | ADP-ribosylation factor 4 | -0.68 |
| GNB2L1 | Guanine nucleotide-binding protein subunit beta-2-like 1;Guanine nucleotide-binding protein subunit beta-2-like 1, N-terminally processed | -0.63 |
| RAN | GTP-binding nuclear protein Ran | -0.63 |
| ALDOA | Fructose-bisphosphate aldolase A | -0.61 |
| MYH9 | Myosin-9 | -0.59 |
| RPSA | 40S ribosomal protein SA | -0.54 |
| FASN | Fatty acid synthase;[Acyl-carrier-protein] S-acetyltransferase;[Acyl-carrier-protein] S-malonyltransferase;3-oxoacyl-[acyl-carrier-protein] synthase;3-oxoacyl-[acyl-carrier-protein] reductase;3-hydroxyacyl-[acyl-carrier-protein] dehydratase;Enoyl-[acyl-carrier-protein] reductase;Oleoyl-[acyl-carrier-protein] hydrolase | -0.52 |
| DCTPP1 | dCTP pyrophosphatase 1 | -0.48 |
| ACAT2 | Acetyl-CoA acetyltransferase, cytosolic | -0.47 |
| PGAM1 | Phosphoglycerate mutase 1 | -0.41 |
| TUBB8 | Tubulin beta-8 chain | -0.40 |
| ANXA2 | Annexin A2 | -0.39 |
| CCT5 | T-complex protein 1 subunit epsilon | -0.37 |
| EEF1A1 | Elongation factor 1-alpha 1 | -0.36 |
| ANXA3 | Annexin A3 | -0.34 |
| HSP90AB1 | Heat shock protein HSP 90-beta | -0.33 |
| NME2;NME2P1 | Nucleoside diphosphate kinase B;Putative nucleoside diphosphate kinase | -0.33 |
| TUBB | Tubulin beta chain | -0.28 |
| TUBB4B | Tubulin beta-4B chain | -0,27 |
| EEF2 | Elongation factor 2 | -0.26 |
| YWHAZ | 14-3-3 protein zeta/delta | -0.23 |
| 6PGD | 6-phosphogluconate dehydrogenase, decarboxylating | -0.19 |
| TPI1 | Triosephosphate isomerase | -0.19 |
| CLIC1 | Chloride intracellular channel protein 1 | -0.18 |
| PPIA | Peptidyl-prolyl cis-trans isomerase A;Peptidyl-prolyl cis-trans isomerase A, N-terminally processed | -0.17 |
